# Assessment of current patient reported outcome measures for three core outcome domains for single-sided deafness device intervention trials

**DOI:** 10.1186/s41687-025-00902-4

**Published:** 2025-06-15

**Authors:** Roulla Katiri, Deborah A. Hall, Derek J. Hoare, Sandra Smith, Bethany Adams, Kathryn Fackrell, Adele Horobin, Nicholas Hogan, Nóra Buggy, Pádraig T. Kitterick

**Affiliations:** 1 Hearing Sciences, Mental Health and Clinical Neurosciences, School of Medicine, University of Nottingham, Nottingham, NG7 2UH UK; 2 NIHR Nottingham Biomedical Research Centre, Ropewalk House, 113 The Ropewalk, Nottingham, NG1 5DU UK; 3 Department of Audiology, Mater Misericordiae University Hospital, Eccles Street, Dublin, D07 R2WY Ireland; 4 Adult Diagnostic Audiology, University College London Hospitals NHS Foundation Trust, Royal National ENT & Eastman Dental Hospitals, London, WC1E 6DG UK; 5 Department of Psychology, School of Social Sciences, Heriot-Watt University, Edinburgh, EH14 4AS UK; 6 Nottingham University Hospitals NHS Trust, Queen’s Medical Centre, Derby Road, Nottingham, NG7 2UH UK; 7 Wessex Institute, University of Southampton, University Road, Southampton, SO17 1BJ UK; 8 National Acoustic Laboratories, Australian Hearing Hub, Macquarie University, 16 University Avenue, Macquarie Park, NSW 2109 Sydney, Australia

**Keywords:** Single-sided deafness, Core outcome domain set, Clinical trials, Outcome measures, Measurement instruments, Patient reported outcome measures

## Abstract

**Background:**

Outcome reporting in clinical trials of auditory interventions for adults with Single-Sided Deafness (SSD) is inconsistent. The Core Rehabilitation Outcome Set for Single-Sided Deafness (CROSSSD) initiative has recommended three outcome domains as a minimum standard in the design of SSD intervention clinical trials. These are, *Spatial orientation, Group conversations in noisy social situations*, and *Impact on social situations*. The study objectives were to (i) understand exactly what the outcome domains mean to SSD experts, and (ii) identify and assess candidate PROMs in terms of how well they measure the experts’ conceptualisation of those SSD outcome domains.

**Methodology:**

Stakeholder representatives participated in two semi-structured online focus groups. Participants were four adults diagnosed with SSD with experience of auditory interventions, two healthcare professionals working in the field, and one clinical researcher with experience in evaluating interventions. Thematic analysis was used to determine conceptual elements of each domain. COnsensus-based Standards for the selection of health Measurement INstruments (COSMIN) initiative recommendations were adopted to assess the relevance and comprehensiveness (content validity) of available candidate instruments.

**Results:**

Multiple key concepts were identified for each outcome domain, and presented as a taxonomy. To be acceptable, any measurement instrument would need to achieve good coverage of all concepts in this taxonomy. From the 76 candidate instruments reviewed, none met accepted standards for content validity for SSD. The best performing candidates were (i) *Spatial orientation*: the Spatial Hearing Questionnaire and two variants of the Speech, Spatial and Qualities scale (SSQ-12, SSQ-18-C), (ii) *Group conversations in noisy situations*: the Communication Profile for Hearing Impaired (CPHI) questionnaire, SSQ-12, SSQ-18-C, and a multi-item questionnaire developed by Schafer and colleagues, and (iii) *Impact on social situations*: the CPHI questionnaire.

**Conclusions:**

Multi-dimensional outcome domains introduce specific considerations for how they should be measured. Although some candidates instruments had reasonable comprehensiveness, modification is needed to ensure that there is overall greater relevance to the key concepts.

**Supplementary information:**

The online version contains supplementary material available at 10.1186/s41687-025-00902-4.

## Background

Single-Sided Deafness (SSD), or unilateral hearing loss, is defined by hearing thresholds within normal limits in one ear, and a severe-to-profound sensorineural hearing loss in the other ear [1]. SSD can be congenital [2–7], or acquired due to conditions such as Ménière’s disease [8], viral infections such as labyrinthitis or COrona VIrus Disease (COVID) [9–11], autoimmune systemic diseases, or vestibular schwannoma [12–15]. In some cases the cause of SSD is unknown [16–20]. It is estimated that SSD affects 12 to 27 individuals in every 100,000 of the general population [21].

SSD poses the listener with significant challenges [22–24]. These include poor speech perception in complex listening environments [12, 25–30] and poor spatial awareness of sounds [22, 31–33]. Associated fatigue, psychological, and social consequences have also been documented [23, 34–41]. Various hearing aids (e.g., contralateral routing of signals), and auditory implants (e.g., bone conduction devices, cochlear implants) have been utilised since the 1960s to alleviate the functional effects of SSD [22, 42–51]. However, choice of outcomes and measurement instruments to assess the benefits and harms of interventions for SSD have been diverse and inconsistent [52, 53]. A systematic review including 96 studies evaluating the therapeutic benefits and harms of SSD interventions identified many ways to measure the same domains of interest. Notably, no single measurement instrument was used by all studies [54]. For example, speech-related outcome domains were measured using 73 different measurement instruments, and spatial-related domains were measured using 43 different measurement instruments. Another systematic review of studies evaluating the effectiveness of SSD interventions identified that outcome selection was biased towards assessing functional impairments for which measures are readily available and widely used, e.g., speech perception testing in noise, and localisation tests [53]. Outcomes that assess an individual’s well-being and overall health are also relevant to those receiving treatment for SSD but these are less often measured [55]. The diverse and inconsistent use of outcome measures hinders our ability to compare or synthesise evidence and make informed decisions about optimal treatment of SSD [56–59].

The ideal scenario is that experts in SSD (healthcare users, healthcare professionals, clinical researchers) are involved in prioritising what treatment-related outcomes are critical and important to measure, so that trialists can select and administer instruments which measure those outcomes and which have good psychometric properties. The Core Rehabilitation Outcome Set for Single-Sided Deafness (CROSSSD) initiative was established to progress towards this goal. To date, the CROSSSD team have employed good practice methodology following Core Outcome Measures in Effectiveness Trials (COMET) initiative recommendations [56] to complete the prioritisation phase. An international Delphi study involving 308 experts in SSD and a meeting with key stakeholder representatives took place in order to reach a consensus [60–64]. The consensus decision was that three outcome domains are critical and important to assess in every SSD clinical trial. These are (i) *Spatial orientation*, (ii) *Group conversations in noisy social situations*, and (iii) *Impact on social situations*. A plain language definition for each of these outcome domains were co-created by the CROSSSD team and two study research partners with lived experience of SSD (Table 1).

**Table 1:** The core outcome domain set for single-sided deafness [68]

Outcome domain name	Plain language definition
Spatial orientation	Knowing where you are in relation to the position of a sound source
Group conversations in noisy social situations	Listening and following a conversation between a group of people, when others are talking in the background
Impact on social situations	Your hearing loss or device limiting your ability to fully participate in the social world; especially in challenging situations or where a lot of effort is needed to follow the conversation (for example; at a restaurant; at the park; in a bar or at a party)

The next phase of the CROSSSD plan is to determine whether any existing outcome instruments appropriately measure *Spatial orientation, Group conversations in noisy social situations*, or *Impact on social situations*. Good practice guidelines exist to guide researchers through the steps to determine what is ‘appropriate’. For example, COSMIN (COnsensus-based Standards for the selection of health Measurement INstruments) guidelines set out a taxonomy of measurement properties relevant for evaluating PROMs; namely validity, reliability, and responsiveness. The COSMIN initiative states that one of the most important psychometric properties of a measurement instrument is its content validity [60, 61]. Content validity refers to the degree to which the items within an instrument are an adequate reflection of the construct to be measured. The measure of content validity encompasses (i) *relevance* (all instrument items are pertinent to the overall concept), and (ii) *comprehensiveness* (the instrument samples all the key elements of the construct) [60, 62].

The objectives of the present study were therefore to (i) understand exactly what the outcome domains mean to SSD experts, and (ii) identify and assess candidate PROMs for their relevance and comprehensiveness in measuring the SSD outcome domains.

## Methods

Our approach involved people who had been treated for SSD (patient and public involvement collaborators NH and NB) as CROSSSD study research partners thus ensuring that decisions were informed by lived experience [63]. Ethical approval was granted from the Proportionate Review Nottingham 2 Research Ethics Committee (REC reference 19/EM/0222, Integrated Research Application System (IRAS project ID 239,750) on 06 August 2019. Informed consent was taken prior to participation at the focus groups using an online consent form. Participants were reminded that they could withdraw from the study at any point without needing to give a reason. All participants were volunteers and no reimbursement was given for their contribution.

### Objective 1. Domain conceptualisation

A thematic approach was taken using two expert focus groups to explore the personal patterns of experience and meaning of the three outcome domains. Focus groups were conducted online in October 2020, using Microsoft Teams software. One week before the focus group, participants received the plain language definition of each outcome domain (Table 1) and were asked to reflect on how the outcome domains related to their personal experiences. Informed consent using an online form was obtained prior to the focus group discussion.

### Participants

Participants were Healthcare Users (HU) diagnosed and treated for SSD, and Healthcare Professionals (HP) working in the field. Inclusion criteria were: (i) adults ≥ 18 years of age that were healthcare users or professionals with experience in the field of SSD, and (ii) had participated in the CROSSSD study consensus meeting [64]. To maximise diversity in expertise and to ensure the focus groups were representative of the consensus meeting experts, purposive sampling targeted a wide range of demographics (gender, age, country, cause of SSD), and experience of different SSD interventions. In total, four HU and three HP were recruited (Table 2). One further HP had to withdraw before the focus group meeting.

**Table 2:** Participant demographics. SSD expertise (for Healthcare Users (HU) this was the number of years since their diagnosis i.e., their lived experience; for Healthcare Professionals (HP) this was the number of years since they started working in the field i.e., their occupational experience); and SSD intervention experience

	Participants	Gender	Age range (years)	Country	SSD expertise	SSD intervention experience
**Focus group 1** Held on 22^nd^ of October 2020	Healthcare User 1 (HU1)	Male	70–79	England	28 years	CROS aid
	Healthcare User 2 (HU2)	Male	60–69	England	3 years, 9 months	CROS aid
	Healthcare Professional 1 (HP1)	Male	50–59	England	35 years	CROS aids
**Focus group 2** Held on 27^th^ of October 2020	Healthcare User 3 (HU3)	Male	18–29	England	1 year, 1 month	CROS aid/BCD/CI
	Healthcare User 4 (HU4)	Female	30–39	Spain	3 years	CROS aid
	Healthcare Professional 2 (HP2)	Male	40–49	Germany	25 years	CROS aids/BCD/CI
	Healthcare Professional 3 (HP3)	Male	60–69	Netherlands	32 years	CROS aids/BCD/CI

(CROS: Contralateral routing of signals aid; BCD: Bone conduction device; CI: Cochlear Implant)

### Data collection instruments and technologies

During the focus groups, facilitators briefly reminded the participants of (i) the meeting ground rules, (ii) the task in hand, in the context of core outcome set development for SSD interventions, and (iii) the plain language definition of each outcome domain. Each outcome domain was allocated 45 minutes for discussion, with a short rest break between each discussion. The *Spatial orientation* domain was discussed first, followed by *Group conversations in noisy social situations*, and *Impact on social situations* last. Each participant was given a clear turn to voice their opinions and time was given prior to closing the discussion to comment on other participants’ views, or add further comments. Participants were given a choice when to take their turn, depending on how prepared they felt to discuss the particular outcome domain.

**Fig. 1:**
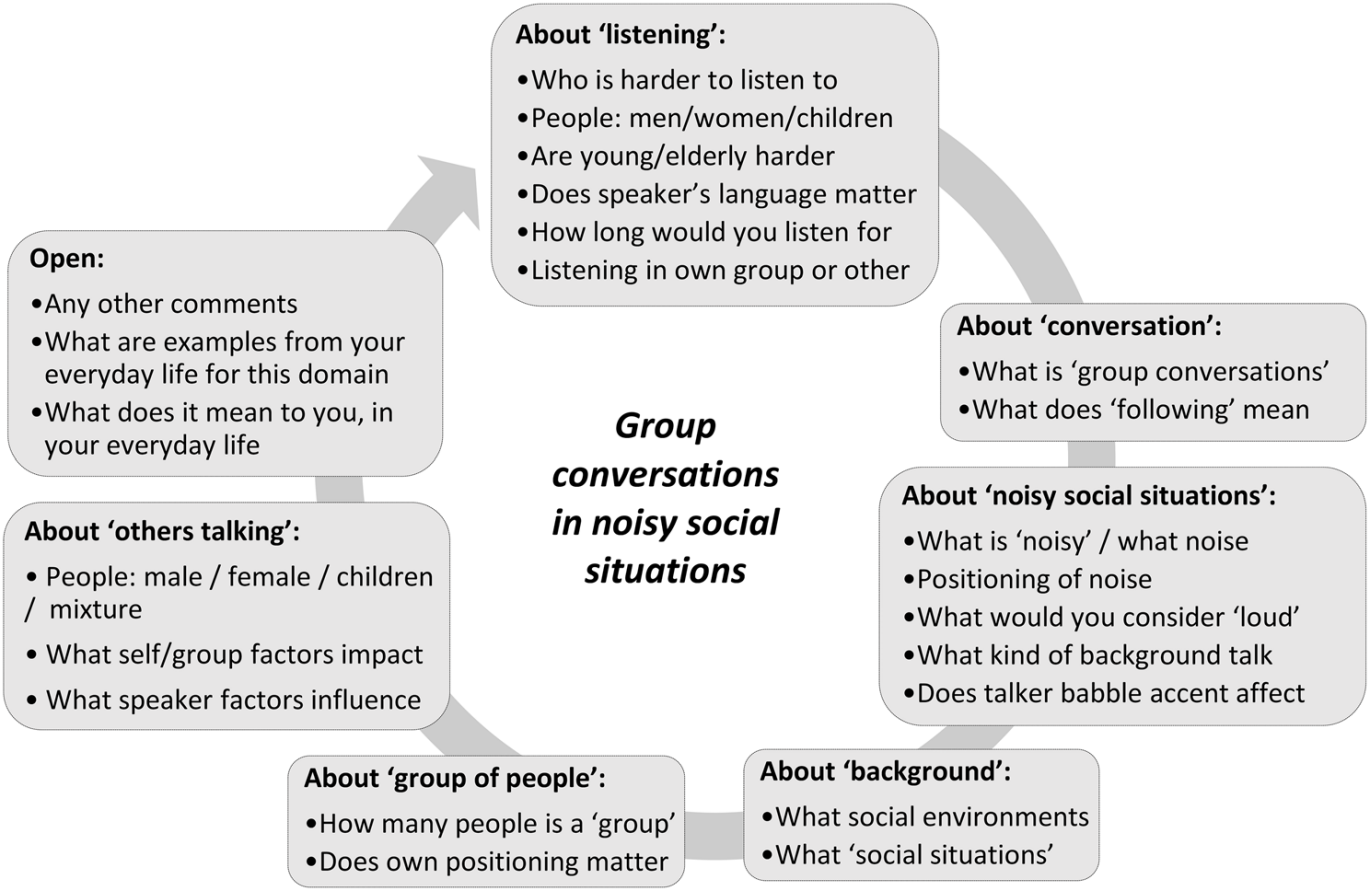
Example of the discussion prompts prepared for the *Group conversations in noisy social situations* (Listening and following a conversation between a group of people; when others are talking in the background) outcome domain to facilitate discussions during the focus groups. Please refer to Additional file 1 for discussion prompts prepared for the other two outcome domains

A semi-structured interview schedule was prepared by RK, PTK, and DAH. The schedule encouraged free narrative responses for analysis using a thematic analysis method [65]. The schedule was reviewed for content, suitability and structure of the prompts, and clarity of the themes by the CROSSSD study research partners (NB, NH) and PPI engagement manager (AH). They ensured that concepts were described clearly, participant material was written in plain language and online communication aids (e.g., captions) were sufficient for participant needs. See Fig. 1 for the schedule used to discuss *Group conversations in noisy social situations*, and refer to Additional file 1 for discussion prompts prepared for the other two outcome domains. The schedule incorporated open-ended questions, designed to elicit detailed descriptions from participants, probed the experience of conversations, the nature of a group, the nature of the background noise, and what in their experience constituted ‘noisy’. The schedule was followed for each outcome domain, for both focus groups.

Facilitators took a position of appreciative inquiry, using active listening and being non-judgemental, and curious to explore and fully understand the participants’ experience. The focus groups were co-facilitated by the lead for the CROSSSD study (RK) who is also a clinical audiologist with 14 years’ clinical experience including working with patients who have SSD, and a researcher (PTK) who has previous qualitative research experience in the field of SSD [35]. The sessions were video-recorded and subsequently transcribed verbatim by RK.

### Data analysis

Transcriptions were independently reviewed by RK and PTK, and key discussion concepts were identified. Initial codes highlighted all keywords and phrases used by participants to describe the different dimensions of each outcome domain. Codes were organised to create a taxonomy of findings, with each outcome domain comprising multiple functional domain topics (such as ‘Knowing where sounds are in relation to you’) which could be further broken down into key concepts (such as ‘Incorporates sound locations that are both in front and behind’ and ‘Considers sounds that are both within and outside the visual field’). Where further clarification was necessary, this coding process referred back to the original transcription. Some codes did not readily fit into the taxonomy and so are reported separately.

Two levels of peer review were conducted to enhance trustworthiness in the taxonomy. First, it was reviewed by the rest of the study team (DAH, DJH) and the CROSSSD study research partners (NH, NB). The study research partners confirmed that the taxonomy captured all important aspects of their lived experience. Second, it was reviewed at an international CROSSSD study steering group meeting, comprising three expert healthcare professionals and researchers based in Europe and the United States, as well as a PPI manager (AH). The individual members of the steering group pre-reviewed the frameworks independently prior to the meeting. Their critical review led to the addition of one key concept for the *Spatial orientation* domain (‘Incorporates sounds that are static or moving’), and participant support for this concept was found in the transcripts of the focus groups.

### Objective 2. Content validity

**Fig. 2:**
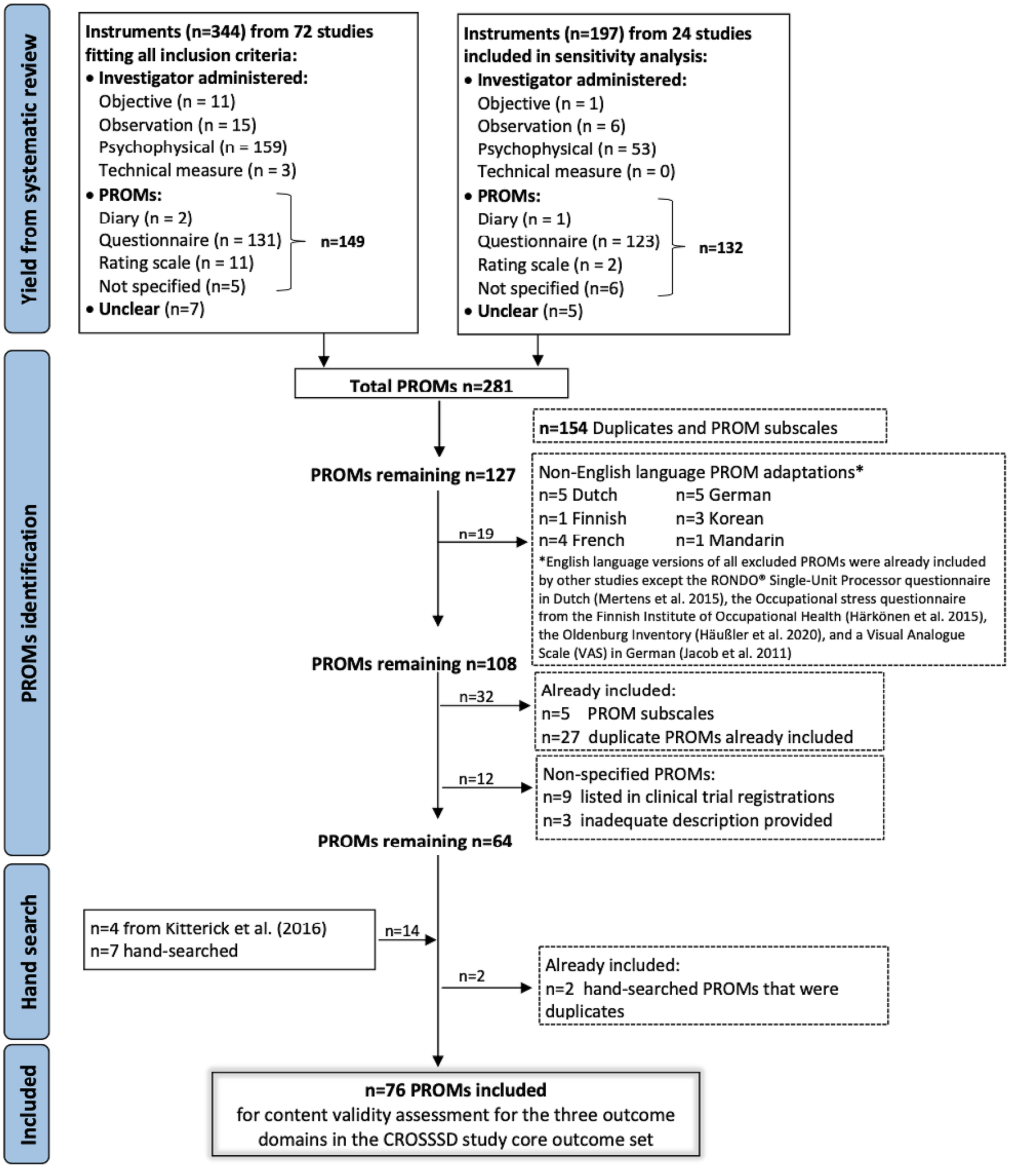
Measurement instruments reported in studies investigating interventions that seek to restore hearing in adults with single-sided deafness. PROM(s): Patient Reported Outcome Measure(s), VAS: Visual analogue scale, CROSSSD: Core Rehabilitation Outcome Set for Single-Sided Deafness, SSD: Single-Sided Deafness

The aim of the next stage was to evaluate whether any existing instruments would be appropriate for measuring the SSD core outcome domain set now that the key concepts within each domain were identified and labelled. Previous research had identified 127 potential instruments for assessing SSD outcomes (Fig. 2). These were two systematic reviews of outcome domains and measurement instruments used in the field of SSD [54], and of hearing instruments for unilateral severe-to-profound sensorineural hearing loss [53]. Screening for English language versions of these instruments, and supplementing the list with a hand search of more recently published instruments, resulted in a final set of 76 candidate instruments (Fig. 2). These comprised two diary records, 49 questionnaires, 17 rating scales, and eight other Patient Reported Outcome Measures (PROMs) (e.g., patient report with yes/no answers, or a single-item instrument). Some of the instruments were specific to SSD (e.g., Bern Benefit in Single-Sided Deafness questionnaire), others were not (e.g., Abbreviated Hearing Aid Benefit Profile). Additional file 2 provides further details about the 76 candidate PROMs.

All 76 candidate PROMs were first assessed for content relevance. Three coders (RK, SS, BA) independently scored each item in every PROM against the key concepts listed in the taxonomy. Coders had expertise in hearing sciences research, had access to this taxonomy (i.e., plain language definition of the core domain, functional domain topics and key concepts), and used Excel spreadsheets to record their scores (one spreadsheet per PROM).

To score item relevance, coders were asked to consider the key concept, the target population of interest (i.e., healthcare users with SSD) and the context of use (i.e., patient reported outcome from an SSD intervention [60]. Coders were advised that an individual item could be relevant for one or more key concepts. Coders considered these three features of the item and gave a single score as follows: 1 = ‘item irrelevant to the key concept’, 2 = ‘item somewhat relevant/possibly relevant to the key concept’, or 3 = ‘item explicitly/clearly relevant to the key concept’. A scoring spreadsheet template was piloted using five PROMs and all coders were thoroughly trained by RK to ensure consistency in coding, and for quality assurance [62, 66].

Agreement that an item was relevant was achieved if at least two out of the three coders scored the item as 3. Agreement that an item was not relevant was achieved if none of the three coders scored the item as 3. For the remaining items, the three coders met up to discuss the rationale for their scores against the above criteria with the aim of achieving reconciliation on the final classification (i.e., relevant/not relevant). For each PROM, three relevance metrics were generated using the agreed classifications; one for the *Spatial orientation* domain, one for the *Group conversations in noisy social situations* domain, and one for *the Impact on social situations* domain. For example, if a PROM had 10 items and all 10 items were classified as relevant for measuring the outcome domain in question, then the relevance metric for the PROM would be 100%. If eight out of 10 items were classified as relevant to that domain, then the relevance metric for the PROM would be 80%, and so on.

Next, all 76 candidate PROMs were assessed for comprehensiveness. Using the agreed classifications for relevance, comprehensiveness was calculated for the two lowest level in the taxonomy (i.e., comprehensiveness in terms of whether the PROM items cover all key concepts). Relevant and/or not relevant classifications were scored as follows: if a core outcome domain contained eight key concepts and all eight key concepts were covered by at least one item in the PROM, then the comprehensiveness metric for the PROM would be 100%. If six out of the eight key concepts were covered by an item, then the comprehensiveness metric for the PROM would be 75%, and so on.

### Protocol variations

There were two amendments to the published protocol [64]. The first was a change from a face-to-face, 7-hour focus group to two 3-hour web-based meetings. This change was necessary because of travel and physical distancing restrictions imposed by the COVID pandemic. The second deviation from protocol was that the ratio of healthcare users to professional experts was 1:1 rather than the 4:1 stated in the protocol. All participants had previous interactions and an established relationship with the facilitators and each other, having met in a previous stage of this work (consensus meeting that took place in July 2020); and a social coffee morning held shortly before the focus groups [67, 68].

## Results

### Objective 1. Domain conceptualisation

The domain-level taxonomy comprising multiple functional domain topics and key concepts are given in Table 3. Each of the three outcome domains, comprised three functional domain topics. For *Spatial orientation*, these were further broken down into eight key concepts, for *Group conversations in noisy social situations* there were nine key concepts, and for *Impact on social situations* there were also nine key concepts. Most functional domain topics and key concepts had supporting codes from both focus groups, indicating data saturation. For transparency, codes that did not readily fit into the taxonomy are reported in Table 3, while Additional file 3 summarises the main discussion points and supporting participant quotes.

**Table 3:** The functional topics and key concepts for each of the three SSD outcome domains (*Spatial orientation, Group conversations in noisy social situations*, and *Impact on social situations*)

Outcome domain and plain language definition	Functional topics within the domain	Key concepts	Codes that did not readily fit into the taxonomy
***Spatial orientation:*** Knowing where you are in relation to the position of a sound source	Being aware that sounds are not in your visual field	Knowing that the sound is not where you are currently looking	o Experiencing the world as 3-dimensional o Sense of ease or comfort o Sense of security o Personal safety aspect Feelings of: o Inadequacy o Frustration o Anxiety o StressFearConstant challenge
		Being aware of threats or harms outside your visual field	
		Not needing to rely on visual cues	
	Knowing where sounds are in relation to you	Incorporates sound locations that are both in front and behind	
		Incorporates sounds that are static, or moving	
		Considers sounds that are both within and outside the visual field	
	Attending to sounds in one location and not at other locations	Ability to attend to sounds in the correct location without a time delay: an active process	
		Ability to attend to sounds in the presence of noise or other distracting sounds: a dynamic process	
***Group conversations in noisy social situations:*** Listening and following a conversation between a group of people, when others are talking in the background	Dynamic involvement	Knowing when someone has started to talk	o Being aware of all conversations taking place o Contributing appropriately at the right time o Having to rely on visual cues o Having to rely on help or hints provided by a partner o Being able to sustain attention for long enough Feelings of: o Being rude o Embarrassment o Being always on ‘high alert’ o Tiredness o Not being included Loneliness
		Being able to tell when someone new starts to contribute to the group conversation	
		Knowing who to listen to within the group	
		Following the thread of the conversation, when someone starts to contribute, and telling is it’s a new conversation	
	Listening in the background of other conversations	Being able to know if the person talking is part of your conversation or another conversation	
		Being able to separate different streams of conversations	
		Maintain and sustain attention in the conversation	
	Conversations in other background noise	Being able to understand what is being said in a noisy environment	
		Being able to resist distracting sounds	
***Impact on social situations:*** Your hearing loss or device limiting your ability to fully participate in the social world; especially in challenging situations or where a lot of effort is needed to follow the conversation	Contributing to social interactions	Knowing when to take your turn	o Demonstrate an understanding of what others are saying o Impact on relationships, work, education, community, society Feelings of: o Inability to contribute o ‘Over-participation’ o LonelinessExhaustion
		Knowing what to do or say when it’s your turn	
		Being able to take turns without relying on visual cues or prompts from others	
	‘Fitting in’ socially	Feeling that you are contributing socially	
		Feeling that you are part of the social group	
		Not having to avail of help from others to participate	
	Ease of participation	Being able to participate without always having to concentrate intensely	
		Being able to sustain participation over time	
		Not having to avoid or withdraw from a situation	

### Objective 2. Content validity

Regarding the coding of content relevance, coders agreed on the item classification of relevant/not relevant for 43 out of the 76 (57%) candidate PROMs, without the need for any reconciliation conversation. Of the remainder, a classification was agreed through discussion among the three coders. From this agreed coding for the 76 candidate instruments, it was noted that 52 instruments contained no items that were relevant for any of the three core outcome domains. These are recorded in Additional file 2 (last column), and covered domains such as device preference, device satisfaction, coping, number of days missed at work, health status, health-related quality of life, service-related issues, mental health and tinnitus. Included in this list were a number of instruments designed to measure amplification-related outcomes (Glasgow Hearing Aid Benefit Profile [69], International Outcome Inventory for Hearing Aids [70], and Satisfaction with Amplification in Daily Life [71]).

For *Spatial orientation*, two variants of the SSQ (SSQ-12 [72] and SSQ-18-C [73]) scored 100% on the rating of comprehensiveness, indicating that items addressed all eight of the key concepts (Table 4). However, the SSQ-12 and SSQ-18-C scored lower on relevance (25% and 29% respectively) because the instruments also contain questions about speech and qualities of hearing. However, taking only the spatial subscale into account, then for the SSQ-12, the three spatial items were 100% relevant and for the SSQ-18-C, the 17 spatial items were 82% relevant to our *Spatial orientation* domain. We observed that comprehensiveness of the SSQ-12 and SSQ-18-C was compromised because measurement of multiple key concepts relied on a single question, such as ‘You are outside. A dog barks loudly. Can you tell immediately where it is, without having to look?’ [72, 73] or ‘You are standing on the footpath of a busy street. Can you hear right away which direction a bus or truck is coming from before you see it?’ [72, 73]. Therefore, another instrument worth considering for assessing the spatial domain is the Spatial Hearing Questionnaire (SHQ) [74]. This scored 88% on the rating of comprehensiveness (it excluded only one key spatial hearing concept, i.e., ability to attend to sounds in the presence of noise or other distracting sounds) and 50% on the rating of relevance (12 out of the 24 items were relevant to spatial hearing). Unlike the SSQ, key concepts were assessed across multiple items.

**Table 4:** Ratings of relevance and comprehensiveness for each of the core outcome domains for SSD. Scoring for the individual key concepts across each candidate measurement instrument can be found in Additional file 4

Instrument	Relevance			Comprehensiveness		
	***Spatial orientation***	***Group conversations in noisy social situations***	***Impact on social situations***	***Spatial orientation***	***Group conversations in noisy social situations***	***Impact on social situations***
01. Abbreviated Hearing Aid Benefit Profile (APHAB)	0	13	0	0	44	0
02. Audio Processor Satisfaction Questionnaire (APSQ)	0	0	13	0	0	33
03. Bern Benefit in Single-Sided Deafness Questionnaire (BBSS)	10	10	0	13	11	0
04. BAHA satisfaction questionnaire (Ghossaini et al, 2010)	0	7	10	0	22	22
07. Client Orientated Scale of Improvement (COSI)	19	13	13	38	11	22
08. Communication profile for hearing impaired (CPHI)	1	8	18	63	100	100
11. Dizziness Handicap Inventory (DHI)	0	0	4	0	0	11
15. Expected Consequences of Hearing aid Ownership (ECHO)	0	0	6	0	0	11
17. Glasgow Benefit Inventory (GBI)	0	0	6	0	0	11
18. Glasgow Health Status Inventory (GHSI)	0	0	6	0	0	11
21. Hearing Handicap Inventory (HHIA)	0	0	12	0	0	33
22. Hearing Implant Sound Quality Index (HISQUI-NL)	5	26	0	25	67	0
24. Hyperacusis Questionnaire (Khalfa et al, 2002)	0	7	0	0	11	0
26. Monaural auditory capacity assessment scale (MACAS)	22	17	0	75	56	0
27. Multi-item, multi-domain questionnaire Schafer et al., 2013) [76]	2	14	2	13	89	11
28. Nijmegen Cochlear Implant Questionnaire (NCIQ)	5	0	5	75	0	22
41. Questionnaire (Snapp et al, 2010)	50	25	0	13	11	0
47. Speech Spatial & Qualities 12 items (SSQ-12)	25	17	8	100	89	11
49. Spatial Hearing Questionnaire (SHQ)	50	0	0	88	0	0
50. Speech, Spatial & Qualities 12 items Comparative (SSQ-12-C)	25	17	8	63	56	11
51. Speech, Spatial & Qualities 12 Pre and Post (SSQ-12-B)	25	17	8	63	56	11
52. Speech, Spatial & Qualities 18 items Comparative (SSQ-18-C)	29	8	0	100	89	0
53. Speech, Spatial & Qualities 5 items (SSQ-5)	20	40	20	50	44	11
58. Tinnitus Handicap Inventory (THI)	0	0	4	0	0	11

For *Group conversations in noisy social situations*, the CPHI questionnaire [75] scored 100% on the rating of comprehensiveness, indicating that items addressed all nine of the key concepts (Table 4). Again, the CPHI scored lower on relevance (8%). We judged that only 12 out of the 145 items were relevant to group conversations in noisy environments. This is not surprising given the CPHI is a multi-dimensional instrument comprising four broad subscales (communication performance, communication environment, communication strategies, and personal adjustment). The SSQ-12 and SSQ-18-C also performed well on comprehensiveness, both scoring 89% (Table 4). In each of these two questionnaires, only one key concept was not addressed; ‘Being able to know if the person talking is part of your conversation or another conversation’ (SSQ-12), and ‘Being able to separate different streams of conversations’ (SSQ-18-C). Relevance scores for *Group conversations in noisy social situations* was disadvantaged because the SSQ is a multi-dimensional instrument. Taking only the speech subscale into account, then for the SSQ-12, the five speech items were 40% relevant and for the SSQ-18-C, the 14 speech items were 29% relevant to our group conversations domain. The multi-item, multi-domain questionnaire developed by Schafer et al. (2013) [76] scored 89% on comprehensiveness. Overall, this instrument scored poorly on relevance (2%) because only six out of 43 items were about group conversations in noisy social situations. For all four of the instruments reported above (i.e., CPHI, SSQ-12, SSQ-18-C, and the questionnaire by Schafer et al. [76]), comprehensiveness was compromised because results were strongly reliant on single items which asked about multiple key concepts, such as ‘You’re at a dinner party with several other people. How often can you carry on a conversation or give and receive information without a great deal of effort?’ [75], ‘You are in a group of about five people in a busy restaurant. You can/cannot see everyone else in the group. Can you follow the conversation?’ [72, 73], and ‘How difficult is it to understand multiple talkers all around you in noise, at work or school?’ [76].

For *Impact on social situations,* the CPHI questionnaire [75] scored 100% on the rating of comprehensiveness, indicating that items addressed all nine of the key concepts (Table 4). Unlike the two other core domains, the comprehensiveness score was not reliant on single questions because there was more of a one-to-one mapping between one question and one key concept. Regarding relevance, 26 out of the 145 items were judged to be relevant to this core domain (18% relevance). This is perhaps not surprising given that the CPHI is a multi-dimensional instrument comprising four broad subscales (communication performance, communication environment, communication strategies, and personal adjustment). No other candidate instrument was deemed to be appropriate for assessing *Impact on social situations* as defined by our expert stakeholders.

## Discussion

This study engaged with stakeholder representatives to gain an in-depth understanding of exactly what the outcome domains mean to SSD experts and identified and assessed candidate PROMs for their relevance and comprehensiveness in measuring the SSD outcome domains. Outcome domains were complex in that they comprised multiple conceptual components. For example, *Spatial orientation* included aspects of being aware that sounds are not in one’s visual field, knowing where sounds are in relation to oneself, and attending to sounds in one location and not at other locations. The key concepts identified by SSD experts in the present study complement previous themes explored through qualitative research with healthcare users with SSD. Notably, Lucas et al. [35] identified communication tactics for aiding group conversations in noisy situations and for enabling full participation in the social world. These include correct positioning in a social setting to favour access to signals, speech, or visual cues to aid the person’s ability to follow conversations and to fully participate in challenging listening environments.

The most parsimonious way of measuring outcomes is that one outcome domain is measured by one instrument (e.g. [77]). However, some outcome developers consider it to be simpler and less burdensome for healthcare users and health systems to utilise a single instrument rather than many individual PROMs (e.g. [78]). In hearing sciences, it has also been traditional to develop multi-dimensional instruments that assess a number of domains at the same time. Where the core outcome domains identified by our consensus methodology [68] do not match the outcome domains considered by the developers of the original instruments, the disadvantage of this approach is obvious. Several candidate instruments scored well in terms of their comprehensiveness because they captured all key concepts of the core domain of interest, but scored poorly in terms of their relevance because many items within the instrument asked about other aspects of hearing which were irrelevant to that core domain. In this regard, none of the 76 candidate instruments met the COSMIN standards for content validity in the context of assessing the three core outcome domains for SSD. Nevertheless, there are some instruments which are worthy of further consideration because they could be modified to enhance their psychometric properties. Some suggestions about future research directions are discussed for each outcome domain in turn.

For assessing *Spatial orientation*, on balance the SHQ [74] and the SSQ-18-C [73] have the greatest potential for modification in order to create acceptable instruments. Twelve items from the SHQ (Items 13, 14, 15, 16, 17, 18, 19, 20, 21, 22, 23, and 24) and 14 items from the SSQ-18-C (Items 15, 18, 19, 20, 21, 22, 23, 24, 25, 26, 27, 29, 30, and 31) were found to be relevant to the key concepts for *Spatial orientation*. Modification would need to consider removing items that are not relevant to *Spatial orientation*, adding items (for the SHQ) asking about the ability to attend to sounds in the presence of noise or other distracting sounds, and rewording items (for the SSQ-18-C) so that items were more clearly relevant to specific key concepts. Considering the psychometric properties of any modified instrument, the last two points are particularly relevant for other important aspects of validity; namely construct and structural validity. Construct validity is the extent to which the PROM accurately measures the intended construct, while structural validity is the degree to which the PROM items and subscales reflect the underlying dimensionality of the construct being measured.

For *Group conversations in noisy social situations*, none of the existing instruments stood out as adequate candidates for measuring this outcome domain. Although instruments had good domain coverage, even speech-related subscales contained items that were not relevant to our domain of interest. Our findings highlight a gap in current practice which could be addressed through the creation of a new instrument that is more parsimonious for assessing *Group conversations in noisy social situations*. The CPHI, SSQ-12, SSQ-18-C and the questionnaire by Schafer et al. [76] all contain items that could be retained in a such new instrument. There are precedents for taking such an approach in the field of hearing sciences. For example, the developers of the Tinnitus Functional Index created this instrument by selecting the 25 best-functioning items from a pool of 175 items that had been harvested from nine widely used tinnitus questionnaires [79].

There is a growing body of evidence that hearing impairment significantly impacts on social well-being including engaging socially and maintaining inter-personal relationships [36, 80–83]. It is perhaps therefore surprising that for assessing *Impact on social situations*, the CPHI questionnaire [75] was the only identified PROM suitable for assessing this outcome domain. Twenty-six items from the SHQ (Items 5, 9, 12, 28, 31, 38, 41, 45, 52, 61, 65, 69, 72, 78, 80, 84, 92, 104, 109, 110, 116, 131, 135, 141, 144, and 145) were found to be relevant to the key concepts that were identified by our SSD experts. Our findings indicate that development of an instrument tailored to assessing social impacts is warranted. But any modification of the CPHI questionnaire to create a more parsimonious measure of social impact would again need to consider removing items that were not relevant to this domain.

### Limitations

One of the study objectives was to identify and assess candidate PROMs for their relevance and comprehensiveness in measuring the three core outcome domains for SSD trials identified by the CROSSSD initiative [68]. These three outcome domains were identified through an international stakeholder involvement. However, for the domain conceptualisation phase of this project most participants were male, white Caucasian, and British. This geographical bias was in part due to the restriction on eligibility to those who had ‘core outcome set literacy’ [84, 85], i.e., who had already been engaged in the CROSSSD study [64]. It is unlikely that this geographical bias majorly impacted on the results because no material differences were noted in the functional domain topics and key concepts highlighted by focus group 1 (all British, male participants) and focus group 2 (other European, predominately male participants).

### Concluding remarks

An ongoing challenge facing researchers, funders, healthcare professionals, and policy makers globally is adoption of translational research. For example, core outcome set uptake in trials contributes to reducing research waste by limiting selective reporting of outcomes and ensuring that results can be effectively compared and combined [86]. A recent review suggests that core outcome set uptake is low in most research areas [87], with most common barriers being not including all relevant stakeholder representatives in the core outcome set development process, which can reduce the generalisability and credibility of the outcome set [86]. Another identified barrier is not making recommendations on measurement instruments for the domains in the core outcome set [87]. The CROSSSD study group have endeavoured to minimise these barriers by using robust stakeholder engagement methods, including study team members with lived experience of SSD and making recommendations on measurement instruments. One suggestion going forward is to conduct a realist evaluation to understand how the research translation process contributes to health system sustainability and value-based healthcare [88]. Williamson et al. [87] suggest a ‘bottom up’ approach to research translation, which can yield positive outcomes across impact domains in a core outcome set, including advancing knowledge, collaboration and capacity building as well as contributing to changes in policy and practice. For example, an approach where core outcome set developers collaborate with key organisations and communities in a specific health area, to identify, tailor, and promote uptake strategies can be helpful. The review by Saldanha et al. [89] suggests that greater adoption of, and reference to, core outcome sets in regulatory guidance documents can encourage clinical researchers to measure and report consistent and agreed outcomes. Joining forces with different working groups (e.g., American Cochlear Implant Alliance Task Force [90]) could help to harmonise recommendations and would serve to complete the CROSSSD study roadmap [64].

## Electronic supplementary material

Below is the link to the electronic supplementary material.

Supplementary Material 1

Supplementary Material 2

Supplementary Material 3

Supplementary Material 4

## Data Availability

The datasets and material used and/or analysed during the current study are available from the corresponding author on reasonable request.

## List of abbreviations

APHAB: Abbreviated Profile of Hearing Aid Benefit
APSQ: Audio Processor Satisfaction Questionnaire
BAHA: Bone Anchored Hearing Aid
BBSS: Bern Benefit in Single-Sided deafness
BRC: Biomedical Research Centre
CI: Cochlear Implant
COMET: Core Outcome Measures in Effectiveness Trials
COSI: Client Orientated Scale of Improvement
COSMIN: Consensus-based Standards for the selection of health Measurement INstruments
COVID: COrona VIrus Disease
CPHI: Communication Profile for Hearing Impaired
CPMS: Central Portfolio Management System
CRN: Clinical Research Network
CROSSSD: Core Rehabilitation Outcome Set for Single-Sided Deafness
DHI: Dizziness Handicap Inventory
ECHO: Expected Consequences of Hearing aid Ownership
GBI: Glasgow Benefit Inventory
GHSI: Glasgow Health Status Inventory
HHIA: Hearing Handicap Inventory for Adults
HISQUI-NL: Hearing Implant Sound Quality Index
HP: Healthcare Professional
HU: Healthcare User
IRAS: Integrated Research Application System
MACAS: Monaural Auditory Capacity Assessment Scale
NCIQ: Nijmegen Cochlear Implant Questionnaire
NIHR: National Institute for Health Research
PPI: Patient and Public Involvement
PROM(s): Patient Reported Outcome Measure(s)
REC: Research Ethics Committee
SHQ: Spatial Hearing Questionnaire
SSD: Single-Sided Deafness
SSQ: Speech, Spatial and Qualities hearing scale
THI: Tinnitus Handicap Inventory
UK: United Kingdom
VAS: Visual Analogue Scale
