# Supplementary material for: Assessment of current patient reported outcome measures for three core outcome domains for single-sided deafness device intervention trials
Source: J Patient Rep Outcomes. 2025 Jun 15;9:68. doi: 10.1186/s41687-025-00902-4 (PMC12167737; doi:10.1186/s41687-025-00902-4)
Supplement: Supplementary file 1 — Supplementary Material 1 [file 41687_2025_902_MOESM1_ESM.docx]

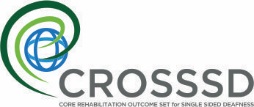


**Additional file 1.** Discussion prompts prepared for the **(a)** *Spatial orientation* and **(b)** *Impact on social situations* outcome domains to facilitate discussions during the focus groups.

**(a)** *Spatial orientation* (Knowing where you are in relation to the position of a sound source).

**(b)** *Impact on social situations* (Your hearing loss or device limiting your ability to fully participate in the social world; especially in challenging situations or where a lot of effort is needed to follow the conversation (for example; at a restaurant; at the park; in a bar or at a party)).

**(c)** *Group conversations in noisy social situations* (Listening and following a conversation between a group of people; when others are talking in the background).
