# Supplementary material for: Assessment of current patient reported outcome measures for three core outcome domains for single-sided deafness device intervention trials
Source: J Patient Rep Outcomes. 2025 Jun 15;9:68. doi: 10.1186/s41687-025-00902-4 (PMC12167737; doi:10.1186/s41687-025-00902-4)
Supplement: Supplementary file 2 — Supplementary Material 2 [file 41687_2025_902_MOESM2_ESM.docx]

**Additional file 2.** Complete list of the 76 candidate Patient Reported Outcome Measures (PROMs) assessed in this study.

| **Instr Ax No** | **Measurement instrument** | **Developers / Reference** | **Year developed** | **PROM type** | **n of items** | **Structure** | **SSD specific?** | **Relevant to core domains?** |
| --- | --- | --- | --- | --- | --- | --- | --- | --- |
| **1** | Abbreviated Hearing Aid Benefit Profile (APHAB) | Cox, R. M., & Alexander, G. C. (1995). The Abbreviated Profile of Hearing Aid Benefit. *Ear Hear*, 16(2), 176–186. doi: 10.1097/00003446-199504000-00005. | 1995 | Questionnaire | 24 | Consists of everyday situations. Requires Pt to circle the answer that is closest to their own experiences A (Always, 995%) to G (Never, 1%) with and without their hearing aid. | No | Yes |
| **2** | Audio Processor Satisfaction Questionnaire (APSQ) | Billinger-Finke, M., Bräcker, T., Weber, A., Amann, E., Anderson, I., & Batsoulis, C. (2020). Development and validation of the audio processor satisfaction questionnaire (APSQ) for hearing implant users. *Int J Audiol*, 59(5), 392–397. doi: 10.1080/14992027.2019.1697830. | 2018? | Questionnaire | 21 | Consists of a 5-point Likert scale with a range from ‘never’ to ‘always’ plus a ‘not applicable’ field. | No | Yes |
| **3** | Bern Benefit in Single-Sided Deafness (BBSS) questionnaire | Kompis, M., Pfiffner, F., Krebs, M., & Caversaccio, M. D. (2011). Factors influencing the decision for Baha in unilateral deafness: The Bern benefit in single-sided deafness questionnaire. *Adv Otorhinolaryngol,* 71, 103–111. doi: 10.1159/000323591. | 2011 | Questionnaire | 10 | Consists of a 11-point Likert scale with a range from -5 ('much better without the aid') to +5 ('much better with the aid'). | Yes | Yes |
| **4** | Bone Anchored Cochlear Stimulator (BAHA) satisfaction questionnaire | Ghossaini, S. N., Spitzer, J. B., & Borik, J. (2010). Use of the Bone-Anchored Cochlear stimulator (Baha) and satisfaction among long-term users. *Semin Hear*, 31(01), 3–14. | 2010 | Questionnaire | 30 | Consists of a 5-point Likert scale with a range from ‘strongly agree’ to ‘strongly disagree’ plus a ‘not applicable’ field. | No | Yes |
| **5** | Brief- Coping Orientation to Problems Experienced (COPE) questionnaire | Carver, C. S. (1997). You want to measure coping but your protocol’s too long: Consider the brief COPE. *Int J Behav Med*, 4(1), 92–100. doi: 10.1207/s15327558ijbm0401_6. | 1997 | Questionnaire | 28 | Consists of a 4-point Likert scale with a range from ‘I haven't been doing this at all’ to ‘I’ve been doing this a lot’. | No | No |
| **6** | Center for Epidemiologic Studies Depression Scale (CES-D) English adaptation of ADS-L | Lewinsohn, P. M., Seeley, J. R., Roberts, R. E., & Allen, N. B. (1997). Center for Epidemiologic Studies Depression Scale (CES-D) as a screening instrument for depression among community-residing older adults. 12(2), 277–287. doi: 10.1037//0882-7974.12.2.277. | 1997 | Questionnaire | 20 | Response options range from 0 to 3 for each item (0 = Rarely or None of the Time, 1 = Some or Little of the Time, 2 = Moderately or Much of the time, 3 = Most or Almost All the Time). Scores range from 0 to 60, with high scores indicating greater depressive symptoms. | No | No |
| **7** | Client Orientated Scale of Improvement (COSI) | Dillon, H., James, A., & Ginis, J. (1997). Client Oriented Scale of Improvement (COSI) and its relationship to several other measures of benefit and satisfaction provided by hearing aids*. J Am Acad Audiol,* 8(1), 27–43. | 1997 | Questionnaire | 16 | Consists of a 5-point Likert scale with a range from ‘worse’ to ‘much better’. Contains a choice of 16 categories. | No | Yes |
| **8** | Communication profile for hearing impaired (CPHI) | Demorest, M. E., & Erdman, S. A. (1987). Development of the communication profile for the hearing impaired. *J Speech Hear Disord*, 52(2), 129–143. doi: 10.1044/jshd.5202.129. | 1987 | Questionnaire | 145 | Consists of 3 parts. Part I covers communication with others (Qn 1-18), Part II covers 'experiences when communicating with others (Qn 19-76). Part III covers 'feelings, attitudes, beliefs' (Qn 77-145). Responses are gathered with a 5-point rating scale. | No | Yes |
| **9** | Diary record: Characterized their tinnitus in a diary. Each day, they were asked to rate the loudness and stress caused by the tinnitus in a visual analogue scale, as well as their mood and their ability to influence the tinnitus. In the evaluation, visual analogue scale was translated to a scale from 0 to 10. The ratings were averaged for each subject over all 4 questions and each month | Buechner, A., Brendel, M., Lesinski-Schiedat, A., Wenzel, G., Frohne-Buechner, C., Jaeger, B., & Lenarz, T. (2010). Cochlear implantation in unilateral deaf subjects associated with ipsilateral tinnitus. *Otol Neurotol*, 31(9), 1381–1385. doi: 10.1097/MAO.0b013e3181e3d353. | 2010 | VAS | 4 | Consists of a 10-point Likert scale from 0 to 10, with 10='better'. Categories were: loudness, stress, mood, ability to influence tinnitus. Ratings of all four categories were averaged monthly. | Not sure | No |
| **10** | Diary record: During this evaluation period, Pt were asked to switch devices daily and to complete a diary in which they had to rate overall satisfaction, clearness of sound (CS), and effort of listening in background noise (BN) on a scale from 1 to 10. Free space was provided for personal comments of the patient | Desmet, J. B. J., Wouters, K., De Bodt, M., & Van de Heyning, P. (2012). Comparison of 2 implantable bone conduction devices in Pt with single-sided deafness using a daily alternating method. *Otol Neurotol*, 33(6), 1018–1026. doi: 10.1097/MAO.0b013e31825e79ba. | 2012 | Diary | 4 | Diary record: During this evaluation period, Pt were asked to switch devices daily and to complete a diary in which they had to rate overall satisfaction, clearness of sound (ranging from 0=unclear to 10=very clear), and effort of listening in background noise (ranging from 0=no effort to 10=very much effort) on a scale from 1 to 10. Free space was provided for personal comments for 9 situations (Positive numbers=improvement, negative numbers=deterioration). | Not sure | No |
| **11** | Dizziness Handicap Inventory (DHI) | Jacobson, G. P., & Newman, C. W. (1990). The development of the Dizziness Handicap Inventory. *Arch Otolaryngol Head Neck Surg*, 116(4), 424–427. doi: 10.1001/archotol.1990.01870040046011. | 1990 | Questionnaire | 25 | Consists of 3 subscales P=Physical, E=Emotional and F=Functional. Pt are presented with questions and asked to mark if they experience it 'Always', 'Sometimes' or 'No'. Top score is 100 (maximum perceived disability). Bottom score is 0 (no perceived disability). | No | Yes |
| **12** | Entific Medical System Questionnaire (EMSQ) | Dutt, S. N., McDermott, A.-L., Jelbert, A., Reid, A. P., & Proops, D. W. (2002). Day to day use and service-related issues with the bone-anchored hearing aid: The Entific Medical Systems questionnaire. *J Laryngol Otol Suppl*, 28, 20–28. doi: 10.1258/0022215021911301. | 2002 | Questionnaire | 13 | Investigates 'Day to day usage', 'wear and tear concerns', 'service related issues' themes. Consists of 2 demographics questions, 8 questions with multiple choice answers, and 3 questions asking for general views on the service, repairs, surgical, nursing, outpatient visits. | No | No |
| **13** | EQ-5D-3L of the EuroQol Group with a Visual Analogue Scale (VAS) | Rabin, R., & de Charro, F. (2001). EQ-5D: A measure of health status from the EuroQol Group. *Ann Med*, 33(5), 337–343. doi: 10.3109/07853890109002087. | 2001 | Questionnaire | 6 | The Pt is presented with 5 headings ('mobility', 'self-care', 'usual activities', 'pain/discomfort' and 'anxiety/depression') and is asked to choose out of 3 answers how their health can be described today. The last Qn is a rating scale of 0 (the worst health you can imagine) to 100 (the best health you can imagine) in 5 point increments and they are asked to write in a box the number on the scale of how their health is today. | No | No |
| **14** | EQ-5D-5L of the EuroQol Group with a Visual Analogue Scale (VAS) v1.2 | Janssen, M. F., Bonsel, G. J., & Luo, N. (2018). Is EQ-5D-5L better than EQ-5D-3L? A head-to-head comparison of descriptive systems and value sets from seven countries. *Pharmacoeconomics*, 36(6), 675–697. doi: 10.1007/s40273-018-0623-8. | 2018 | Questionnaire | 6 | The Pt is presented with 5 headings ('mobility', 'self-care', 'usual activities', 'pain/discomfort' and 'anxiety/depression') and is asked to choose out of 5 answers how their health can be described today. The last Qn is a rating scale of 0 (the worst health you can imagine) to 100 (the best health you can imagine) in 5 point increments and they are asked to write in a box the number on the scale of how their health is today. | No | No |
| **15** | Expected Consequences of Hearing aid Ownership (ECHO) | Cox, R. M., & Alexander, G. C. (2000). Expectations about hearing aids and their relationship to fitting outcome*. J Am Acad Audiol,* 11(7), 368–382; quiz 407. | 2000 | Questionnaire | 18 | Consists of statements about hearing aids. Pt are asked to circle on a 7-point scale the letter A (not at all') to G ('tremendously') that indicates the extent to which they agree with each statement. | No | Yes |
| **16** | Generalized Anxiety Disorder questionnaire (GAD-7) | Spitzer, R. L., Kroenke, K., Williams, J. B. W., & Löwe, B. (2006). A brief measure for assessing generalized anxiety disorder: The GAD-7. *Arch Intern Med,* 166(10), 1092–1097. doi: 10.1001/archinte.166.10.1092. | 2006 | Questionnaire | 8 | Consists of 2 Qn. Qn 1 asks over the last 2wk how often have they been bothered by a series of 7 problems. Pt rate the 7 problems on a 4-point scale of 0 ('not at all sure') to 3 ('nearly every day'). Qn 2 asks to rate impact on work, home, and people on a 4-point scale of 0 'not difficult at all' to 3 'extremely difficult'. A score of 10 or higher means significant anxiety is present. Score over 15 are severe. | No | No |
| **17** | Glasgow Benefit Inventory (GBI) | Robinson, K., Gatehouse, S., & Browning, G. G. (1996). Measuring patient benefit from otorhinolaryngological surgery and therapy. *Ann Otol Rhinol Laryngol*, 105(6), 415–422. doi: 10.1177/000348949610500601. | 1996 | Questionnaire | 18 | Consists of 18 change in health status Qn which assess how the intervention has altered their QoL. The response to each Qn is based on a 5-point Likert scale (1='much worse' to 5='much better'), ranging from a large deterioration in health status through to a large improvement in health status. | No | Yes |
| **18** | Glasgow Health Status Inventory (GHSI) | Hawthorne, G., & Hogan, A. (2002). Measuring disability-specific patient benefit in cochlear implant programs: Developing a short form of the Glasgow Health Status Inventory, the Hearing Participation Scale*. Int J Audiol,* 41(8), 535–544. doi: 10.3109/14992020209056074. | 2002 | Questionnaire | 18 | Consists of 18 health status Qn which ask specific Qn about how the health problem has affected their QoL. The response to each Qn is based on a 5-point Likert scale (1=''frequently or all of the time' to 5='never'), ranging from 'high health status' through to 'low health status'. It has 3 subscales: general (12 Qn), social support (3 Qn) and physical health (3 Qn). Scores range from 0 to +100. | No | Yes |
| **19** | Glasgow Hearing Aid Benefit Profile (GHABP) | Gatehouse, S. (1999). Glasgow hearing aid benefit profile: Derivation and validation of a client-centered outcome measure for hearing aid services. *J Am Acad Audiol*, 10, 80–103. | 1999 | Questionnaire | 5 | Consists of 4 everyday situations that can lead to difficulty with hearing. The Pt is asked to rate them on a 6-point Likert scale (0=N/A, 1='no difficulty' to 5='cannot manage at all') in terms of difficulty, worry, proportion of the time wearing their hearing aid, how much the hearing aid helps, and satisfaction with aid. Pt can also nominate up to 4 new situations in which it is important for them to be able to hear as well as possible and rate them as above. | No | No |
| **20** | Health Utilities Index Mark 3 (HUI-3) | Furlong, W. J., Feeny, D. H., Torrance, G. W., & Barr, R. D. (2001). The Health Utilities Index (HUI) system for assessing health-related quality of life in clinical studies. *Ann Med*, 33(5), 375–384. doi: 10.3109/07853890109002092. | 2001 | Questionnaire | 11 | Asks about various aspects of your health. Pt are asked to think about their health and ability to do things on a day-to-day basis, during the past 4wks. Each Qn has up to 6 multiple-choice answers. The Pt is asked to choose 1. Qn 11 is a QoL rating scale with 10-point increments from 0 ('worst imaginable quality of life') to 100 ('best imaginable quality of life'). The Pt is asked to mark anywhere on the scale how good or bad their overall QoL is. | No | No |
| **21** | Hearing Handicap Inventory (HHIA) | Newman, C. W., Weinstein, B. E., Jacobson, G. P., & Hug, G. A. (1991). Test-retest reliability of the hearing handicap inventory for adults. *Ear Hear*, 12(5), 355–357. doi: 10.1097/00003446-199110000-00009. | 1991 | Questionnaire | 25 | Pt is presented with everyday situations and is asked to check 'Yes' (=4 points), 'Sometimes' (=2 points) or 'No' (=0 points) for the way they hear without a hearing aid. Scored for 'social', 'emotional' and 'total'. Score of 0=no handicap, to 100=total handicap. Percentage of 0-16%=no handicap, 18-42%=mild/moderate handicap and 44%+=significant handicap. | No | Yes |
| **22** | Hearing Implant Sound Quality Index (HISQUI-NL) | Amann, E., & Anderson, I. (2014). Development and validation of a questionnaire for hearing implant users to self-assess their auditory abilities in everyday communication situations: The Hearing Implant Sound Quality Index (HISQUI19). *Acta Otolaryngol,* 134(9), 915–923. doi: 10.3109/00016489.2014.909604. | 2014 | Questionnaire | 19 | Provides subjective feedback about sound quality of hearing implants experienced by the user. | No | Yes |
| **23** | Hospital Anxiety and Depression Scale (HADS) | Zigmond, A. S., & Snaith, R. P. (1983). The hospital anxiety and depression scale. *Acta Psychiatr Scand*, 67(6), 361–370. doi: 10.1111/j.1600-0447.1983.tb09716.x. | 1983 | Questionnaire | 14 | Contains two 7-item scales: one for anxiety and one for depression both with a score range of 0-21. | No | No |
| **24** | Hyperacusis Questionnaire (Khalfa et al, 2002) | Khalfa, S., Dubal, S., Veuillet, E., Perez-Diaz, F., Jouvent, R., & Collet, L. (2002). Psychometric normalization of a hyperacusis questionnaire. *ORL J Otorhinolaryngol Relat Spec*, 64(6), 436–442. doi: 10.1159/000067570. | 2002 | Questionnaire | 14 | Assesses sensitivity to sound and other noises in the environment. Pt is asked to mark out of 4 answers ('no'=0, 'yes a little'=1, 'yes quite a lot'=2, 'yes a lot'=3) which one best applies to them. Total scores range from 0-45, higher scores representing greater hypersensitivity. | No | Yes |
| **25** | International Outcome Inventory for Hearing Aids (IOI-HA) | Cox, R. M., & Alexander, G. C. (2002). The International Outcome Inventory for Hearing Aids (IOI-HA): Psychometric properties of the English version. *Int J Audiol,* 41(1), 30–35. doi: 10.3109/14992020209101309. | 2002 | Questionnaire | 7 | Consists of general Qn and situations. Pt is asked to choose out of 5 answers which best describes their experience with their hearing aids. | No | No |
| **26** | Monaural auditory capacity assessment scale (MACAS) | McLeod, B., Upfold, L., & Taylor, A. (2008). Self reported hearing difficulties following excision of vestibular schwannoma*. Int J Audiol*, 47(7), 420–430. doi: 10.1080/14992020802033083. | 2008 | Questionnaire | 18 | Asks some demographic Qn. Then the Pt is presented with a number of Qn on the difficulties they may be experiencing with their hearing. For each Qn the Pt is asked to circle the number in an 11-point Likert scale of 0 ('not at all') to 10 ('perfectly). The last Qn presents 4 types of hearing difficulty and the Pt is asked to rank them in order of importance to them (1='most important' to 4 'least important'). | Yes | Yes |
| **27** | Multi-item, multi-domain questionnaire (author's own) | Schafer, E. C., Baldus, N., D’Souza, M., Algier, K., Whiteley, P., & Hill, M. (2013). Behavioral and subjective performance with digital CROS / BiCROS hearing instruments. 46, 62–93. | 2013 | Questionnaire | 43 | Pt are presented with listening conditions (hearing at home, hearing at work or school, hearing in social situations, satisfaction and instrument). They are asked to circle on a 7-point Likert scale of 0 ('can function fine') to 6 ('cannot function at all') the level of difficulty they have in each condition with no hearing instrument and with a (Bi)CROS instrument. The last section (Qn 30-43) collects general information about the hearing aid. | Yes | Yes |
| **28** | Nijmegen Cochlear Implant Questionnaire (NCIQ) | Hinderink, J. B., Krabbe, P. F., & van den Broek, P. (2000). Development and application of a health-related quality-of-life instrument for adults with cochlear implants: The Nijmegen cochlear implant questionnaire. *Otolaryngol Head Neck Surg*, 123(6), 756–765. doi: 10.1067/mhn.2000.108203. | 2000 | Questionnaire | 60 | Pt are presented with situations and they are asked to choose out of 5 possibilities 'never' to 'always' how they hear with their CI. A 'not applicable' choice is also available. Qn 56-60 have 5 different categories 'no' to 'quite well', with a 'not applicable' choice too. Situations include physical, psychological, and social. | No | Yes |
| **29** | Number of days the patient has missed work: Data linked to the number of days the patient has missed work will also be gathered from the French health insurance databases | Marx, M., Costa, N. N., Lepage, B., Taoui, S., Molinier, L., Deguine, O., & Fraysse, B. (2019). Cochlear implantation as a treatment for single-sided deafness and asymmetric hearing loss: a randomized controlled evaluation of cost-utility. *BMC Ear Nose Throat Disord*, 19(1), 1. doi: 10.1186/s12901-019-0066-7. | 2019 | Diary | 1 | Counted the number of days missed work. | No | No |
| **30** | Numeric rating scale (0-10): Tinnitus loudness rated by the patient on a 0-10 scale | Lee, D. J. (2015). Cochlear implantation for treatment of single-sided deafness. ClinicalTrials.Gov. https://clinicaltrials.gov/ct2/show/record/NCT02532972 | 2015 | Numeric rating scale | 1 | Tinnitus loudness rated by the patient on a 0-10 scale (no more info). | Not sure | No |
| **31** | Numeric rating scale (max score 10): Tinnitus loudness on a numeric rating scale | Song, J.-J., Punte, A. K., De Ridder, D., Vanneste, S., & Van de Heyning, P. (2013). Neural substrates predicting improvement of tinnitus after cochlear implantation in Pt with single-sided deafness. *Hear Res*, 299, 1–9. doi: 10.1016/j.heares.2013.02.001. | 2013 | Numeric rating scale | 1 | Tinnitus loudness on a numeric rating scale with max score of 10 (no more details). | Not sure | No |
| **32** | Numeric rating scale: Tinnitus loudness on a numeric rating scale | Song, J.-J., Kim, K., Sunwoo, W., Mertens, G., Van de Heyning, P., De Ridder, D., Vanneste, S., Lee, S.-Y., Park, K.-J., Choi, H., & Choi, J.-W. (2017). A quantitative electroencephalography study on cochlear implant-induced cortical changes in single-sided deafness with tinnitus. *Front Hum Neurosci*, 11, 210. doi: 10.3389/fnhum.2017.00210. | 2017 | Numeric rating scale | 1 | Tinnitus loudness on a numeric rating scale with max score of 10 (no more details). | Not sure | No |
| **33** | Numerical rating scales (author's own): The specific questions included ease of use, local discomfort or pain, accidental uncoupling, and user friendliness and were evaluated on a Likert scale ranging from 1 (deterioration) to 5 (improvement) | Leterme, G., Bernardeschi, D., Bensemman, A., Coudert, C., Portal, J. J., Ferrary, E., Sterkers, O., Vicaut, E., Frachet, B., & Grayeli, A. B. (2015). Contralateral routing of signal hearing aid versus transcutaneous bone conduction in single-sided deafness. *Audiol Neurotol*, 20(4), 251–260. doi: 10.1159/000381329. | 2015 | Numeric rating scale | 4 | Consists of a 5-point scale from 0 to 5, with 1='deterioration', and 5='improvement': Questions included ease of use, local discomfort or pain, accidental uncoupling, and user friendliness. | Not sure | No |
| **34** | Patient report (parasthesia / dysesthesia) | Nevoux, J., Coudert, C., Boulet, M., Czajka, C., Tavernier, L., Daval, M., Ayache, D., Meller, R., Rossetto, S., Papon, J. F., & Deveze, A. (2018). Transcutaneous BAHA Attract system: Long-term outcomes of the French multicenter study. *Clin Otolaryngol*, 43(6), 1553–1559. doi: 10.1111/coa.13214. | 2018 | Single question | 1 | Pt reported parasthesia. | No | No |
| **35** | Patient report of hours / day | Newman, C. W., Sandridge, S. A., & Wodzisz, L. M. (2008). Longitudinal benefit from and satisfaction with the Baha system for Pt with acquired unilateral sensorineural hearing loss. *Otol Neurotol*, 29(8), 1123–1131. doi: 10.1097/MAO.0b013e31817dad20. | 2008 | Diary | 2 | Pt recorded in a diary the number of hours/ day and the number of hours/week they used the device over a period of 18 months. | Not sure | No |
| **36** | Patient report of tinnitus relief or severity | Sladen, D. P., Frisch, C. D., Carlson, M. L., Driscoll, C. L. W., Torres, J. H., & Zeitler, D. M. (2017). Cochlear implantation for single-sided deafness: A multicenter study. *Laryngoscope*, 127(1), 223–228. doi: 10.1002/lary.26102. | 2017 | Single question | 1 | Subjective presence of tinnitus was recorded pre- and postoperatively ('improvement' or 'no change' in tinnitus after surgery). | Not sure | No |
| **37** | Patient report: Patient reported use of their BAHA processor (hours / day) | Wazen, J. J., Spitzer, J. B., Ghossaini, S. N., Fayad, J. N., Niparko, J. K., Cox, K., Brackmann, D. E., & Soli, S. D. (2003). Transcranial contralateral cochlear stimulation in unilateral deafness. *Otolaryngol Head Neck Surg*, 129(3), 248–254. doi: 10.1016/S0194-5998(03)00527-8. | 2003 | Questionnaire | 9 | Pt are asked to choose out of 5 answers (more than 8hr, 4-8hr, 2-4hr, less than 2hr) how many hours per day they use their device. | Not sure | No |
| **38** | Perceived Stress Questionnaire (PSQ) | Levenstein, S., Prantera, C., Varvo, V., Scribano, M. L., Berto, E., Luzi, C., & Andreoli, A. (1993). Development of the Perceived Stress Questionnaire: A new tool for psychosomatic research. *J Psychosom Res*, 37(1), 19–32. doi: 10.1016/0022-3999(93)90120-5. | 1993 | Questionnaire | 30 | Pt are presented with sentences and they are asked to circle the number 1 ('almost never'), 2 ('sometimes', 3 ('often') or 4 ('usually') how often it applies to them in general during the last year or two. A 'recent' version asks the same Qn but asks Pt to 'consider only the last month'. PSQ Score = (Raw score -30) / 90. | No | No |
| **39** | Qn: 'How many years of your life would you exchange for getting normal hearing in your deaf ear?' | Louza, J., Hempel, J. M., Krause, E., Berghaus, A., Müller, J., & Braun, T. (2017). Patient benefit from cochlear implantation in single-sided deafness: A 1-year follow-up. *Eur Arch Otorhinolaryngol,* 274(6), 2405–2409. doi: 10.1007/s00405-017-4511-1. | 2017 | Single question | 1 | Pt were asked 'How many years of your life would you exchange for getting normal hearing in your deaf ear?' The Burstrom et al (2007) time trade-off method for determining quality adjusted life years (QUALY.) was used to calculate the results | Not sure | No |
| **40** | Questionnaire (author's own) | Härkönen, K., Kivekas, I., Kotti, V., Sivonen, V., & Vasama, J.-P. (2017). Hybrid cochlear implantation: quality of life, quality of hearing, and working performance compared to Pt with conventional unilateral or bilateral cochlear implantation. *Eur Arch Oto-Rhino-Laryngology*, 274(10), 3599–3604. doi: 10.1007/s00405-017-4690-9. | 2017 | Questionnaire | 6 | Pt are presented with Qn addressing their working performance with the CI. They can choose out of 5 answers ('very much', 'moderately', 'a little', 'no change' and 'worsened' / 'decreased activity' / 'increased fatigue'. | No | No |
| **41** | Questionnaire (author's own) | Snapp, H. A., Fabry, D. A., Telischi, F. F., Arheart, K. L., & Angeli, S. I. (2010). A clinical protocol for predicting outcomes with an implantable prosthetic device (Baha) in Pt with single-sided deafness*. J Am Acad Audiol*, 21(10), 654–662. doi: 10.3766/jaaa.21.10.5. | 2010 | Questionnaire | 4 | Pt are presented with situations regarding speech or direction of sounds and are asked to rate them by choosing one of 5 answers ('always', 'most of the time', 'occasionally', 'seldom' and 'never'). The Pt is also asked to mark if answering the Qn pre-op, post-op for Rt or Lt ear. | Yes | Yes |
| **42** | Questionnaire about the use of the system (author's own): The custom-made questionnaire regarding the use of the adhesive hearing system was used to assess the following specific topics: 1) ‘‘How often did you need to change the adhesive adaptor?,’’ 2) ‘‘Did you experience feedback?,’’ 3) ‘‘Did the adhesive adaptor fall off during normal use?,’’ 4) ‘‘Did you experience skin irritation?,’’ 5) ‘‘How do you rate the sound quality?,’’ 6) ‘‘How do you rate the appearance of the hearing system?,’’ 7) ‘‘During the trial, was the hearing system a useful hearing tool for you?’’ | Mertens, G., Gilles, A., Bouzegta, R., & Van de Heyning, P. (2018). A prospective randomized crossover study in single sided deafness on the new non-invasive adhesive bone conduction hearing system*. Otol Neurotol*, 39(8), 940–949. doi: 10.1097/MAO.0000000000001892. | 2018 | Questionnaire | 7 | Pt were asked regarding the use of the adhesive hearing system during the their trial using these Qn: 1) 'How often did you need to change the adhesive adaptor?', 2) 'Did you experience feedback?', 3) 'Did the adhesive adaptor fall off during normal use?', 4) 'Did you experience skin irritation?', 5) 'How do you rate the sound quality?', 6) ‘‘How do you rate the appearance of the hearing system?', 7) 'During the trial, was the hearing system a useful hearing tool for you?' | Not sure | No |
| **43** | Questionnaire concerning Phonak Audeo Smart IX model CROS | Busk Linnebjerg, L., & Wetke, R. (2014). The benefits of CROS aids for individuals with unilateral sensorineural hearing loss. *Hear* *Balanc Commun*, 12(1), 36–40. doi: 10.3109/21695717.2013.794593. | 2014 | Questionnaire | 37 | Pt are presented with general Qn about their hearing, a few are open, others have a choice of answers. The second part has 11-point Likert point rating scales of 0 ('very poor') to 10 ('very good') where the Pt is asked to rate the use of aids in various situations. The option to comment is also available. | Not sure | No |
| **44** | Satisfaction with Amplification in Daily Life (SADL) | Cox, R. M., & Alexander, G. C. (2001). Validation of the SADL questionnaire. *Ear Hear*, 22(2), 151–160. doi: 10.1097/00003446-200104000-00008 | 2001 | Questionnaire | 15 | Pt are presented with a list of Qn and are asked about their opinions about their hearing aids. For each Qn they are asked to circle one out of 7 letters A ('not at all') to G ('tremendously') that represents the best answer for them regarding the hearing aids they are wearing now. | No | No |
| **45** | Short Form Health Survey (SF-36) | Ware, J. E. (1999). SF-36 Health Survey. In M. E. Maruish (Ed.), The use of psychological testing for treatment planning and outcomes assessment. Lawrence Erlbaum Associates Publishers. | 1999 | Questionnaire | 36 | Pt are presented with general health Qn, Qn about activities, physical health, emotional problems. Each Qn has multiple choice answers ranging from 2 to 6 possible answers. A scoring tool is available. | No | No |
| **46** | Short Tinnitus Questionnaire (Goebel & Hiller) | Goebel, G., & Hiller, W. (1994). The tinnitus questionnaire. A standard instrument for grading the degree of tinnitus. Results of a multicenter study with the tinnitus questionnaire. *HNO*, 42(3), 166–172. | 1994 | Questionnaire | 33 | Consists of 4 subscales (distress and intrusiveness, sleep disturbances, auditory perceptual difficulties, irrational beliefs. Scoring is out of 3 answers 'true', 'partly true' or 'not true'. Normative data is provided. | No | No |
| **47** | Short version of the Speech, Spatial and Qualities (SSQ-12) scale | Noble, W., Jensen, N. S., Naylor, G., Bhullar, N., & Akeroyd, M. A. (2013). A short form of the Speech, Spatial and Qualities of Hearing scale suitable for clinical use: The SSQ12*. Int J Audiol,* 52(6), 409–412. doi: 10.3109/14992027.2013.781278. | 2013 | Questionnaire | 12 | Consist of Qn about aspects of Pt's ability and experience hearing and listening in different situations. Pt are asked to mark on an 11-point Likert scale of 0 ('quite unable to do or experience what is described') to 10 ('would be perfectly able to do or experience what is described'). Pt have the choice to mark 'not applicable'. | No | Yes |
| **48** | Single feedback question (author's own): Upon completion of the study, Pt were asked, 'Taking everything into consideration, would you do it again? That is, would you still proceed with the Baha?' | Newman, C. W., Sandridge, S. A., & Wodzisz, L. M. (2008). Longitudinal benefit from and satisfaction with the Baha system for Pt with acquired unilateral sensorineural hearing loss. *Otol Neuro,* 29(8), 1123–1131. doi: 10.1097/MAO.0b013e31817dad20. | 2008 | Single question | 1 | Pt were asked at the end of the study: 'Taking everything into consideration, would you do it again? That is, would you still proceed with the Baha?' (no more details) | Not sure | No |
| **49** | Spatial Hearing Questionnaire (SHQ) | Tyler, R. S., Perreau, A. E., & Ji, H. (2009). Validation of the Spatial Hearing Questionnaire. *Ear Hear*, 30(4), 466–474. doi: 10.1097/AUD.0b013e3181a61efe. | 2009 | Questionnaire | 24 | Pt are presented with situations and they are asked to respond to each Qn with a number from 0 ('very difficult') to 100 ('very easy'). | Yes | Yes |
| **50** | Speech, Spatial and Qualities 12 Comparative (SSQ-12-C) | Gatehouse, S., & Noble, W. (2004). The Speech, Spatial and Qualities of hearing scale (SSQ*). Int J Audiol*, 43(2), 85–99. doi: 10.1080/14992020400050014. | 2004 | Questionnaire | 12 | Consist of Qn about aspects of the Pt's ability and experience hearing and listening in different situations. Pt are asked to compare their ability and experience with their current aids vs their previous aids. Pt are asked to mark on an 11-point Likert scale of -5 ('things are much worse') through 0 ('things are no different') to +5 ('if things are much better'). Pt have the choice to mark 'not applicable'. | No | Yes |
| **51** | Speech, Spatial and Qualities 12 of Hearing Scale for Benefit Questionnaire (SSQ-12-B) pre and post | Gatehouse, S., & Noble, W. (2004). The Speech, Spatial and Qualities of hearing scale (SSQ*). Int J Audiol*, 43(2), 85–99. doi: 10.1080/14992020400050014. | 2004 | Questionnaire | 12 | Consist of Qn about aspects of the Pt's ability and experience hearing and listening in different situations. Pt are asked to compare their ability and experience with their current aids vs before getting the aids. Pt are asked to mark on an 11-point Likert scale of -5 ('things are much worse') through 0 ('things are no different') to +5 ('if things are much better'). Pt have the choice to mark 'not applicable'. | No | Yes |
| **52** | Speech, Spatial and Qualities 18 Comparative (SSQ-18-C) | Gatehouse, S., & Noble, W. (2004). The Speech, Spatial and Qualities of hearing scale (SSQ). *Int J Audiol*, 43(2), 85–99. doi: 10.1080/14992020400050014. | 2004 | Questionnaire | 49 | Consist of Qn about aspects of the Pt's ability and experience hearing and listening in different situations. Pt are asked to compare their ability and experience with their current aids vs their previous aids. Pt are asked to mark on an 11-point Likert scale of -5 ('things are much worse') through 0 ('things are no different') to +5 ('if things are much better'). Pt have the choice to mark 'not applicable'. | No | Yes |
| **53** | Speech, Spatial, and Qualities of Hearing Scale 5 Questions (SSQ-5) | Gatehouse, S., & Noble, W. (2004). The Speech, Spatial and Qualities of hearing scale (SSQ). *Int J Audiol*, 43(2), 85–99. doi: 10.1080/14992020400050014. | 2004 | Questionnaire | 5 | Consist of Qn about aspects of the Pt's ability and experience hearing and listening in different situations. Pt are asked to compare their ability and experience with their current aids vs their previous aids. Pt are asked to mark on an 11-point Likert scale of -5 ('things are much worse') through 0 ('things are no different') to +5 ('if things are much better'). Pt have the choice to mark 'not applicable'. | No | Yes |
| **54** | Subjective Tinnitus Severity Scale (STSS) | van Veen, E. D., Jacobs, J. B., & Bensing, J. M. (1998). Assessment of distress associated with tinnitus. *J Laryngol Otol,* 112(3), 258–263. doi: 10.1017/s002221510015830x. | 1998 | Questionnaire | 16 | Pt are presented with Qn about the severity of their tinnitus and the related distress. They respond with a Yes/No. | No | No |
| **55** | Time trade off (not specified) comprises one question about how many years of their lives Pt would sacrifice for living with perfect hearing for the rest of their lives. TTO (%) = ((life expectancy – number of years to give up for perfect hearing) / life expectancy) * 100 | Peters, J. P., van Zon, A., Smit, A. L., van Zanten, G. A., de Wit, G. A., Stegeman, I., & Grolman, W. (2015). CINGLE-trial: cochlear implantation for siNGLE-sided deafness, a randomised controlled trial and economic evaluation. *BMC Ear Nose Throat Disord,* 15, 3. doi: 10.1186/s12901-015-0016-y. | 2015 | Single question | 1 | Pt were asked 'How many years of their lives would they sacrifice for living with perfect hearing for the rest of their lives' during the baseline and follow up visits. | Not sure | No |
| **56** | Tinnitus Burden Questionnaire (TBQ): A self-developed questionnaire assessing various aspects of tinnitus burden. It consists of 12 visual analogue scales (VAS), ranging from ‘0’ (no tinnitus burden) to ‘10’ (maximum tinnitus burden) | Peters, J. P., van Zon, A., Smit, A. L., van Zanten, G. A., de Wit, G. A., Stegeman, I., & Grolman, W. (2015). CINGLE-trial: cochlear implantation for siNGLE-sided deafness, a randomised controlled trial and economic evaluation. *BMC Ear Nose Throat Disord,* 15, 3. doi: 10.1186/s12901-015-0016-y. | 2015 | Questionnaire | 12 | Pt are asked Qn about various aspects of tinnitus burden. It consists of 12 visual analogue scales, ranging from 0 ('no tinnitus burden') to 10 ('maximum tinnitus burden'). | Not sure | No |
| **57** | Tinnitus Functional Index (TFI) | Meikle, M. B., Henry, J. A., Griest, S. E., Stewart, B. J., Abrams, H. B., McArdle, R., Myers, P. J., Newman, C. W., Sandridge, S., Turk, D. C., Folmer, R. L., Frederick, E. J., House, J. W., Jacobson, G. P., Kinney, S. E., Martin, W. H., Nagler, S. M., … Vernon, J. A. (2012). The Tinnitus Functional Index: Development of a new clinical measure for chronic, intrusive tinnitus. *Ear Hear,* 33(2), 153–176. doi: 10.1097/AUD.0b013e31822f67c0. | 2012 | Questionnaire | 25 | Pt are presented with Qn about their tinnitus and are asked to rate on an 11-point Likert scale of 0 to 10 or 10% to 100%. Includes 8 subscales (intrusiveness, sense of control, cognitive, sleep, auditory, relaxation, QoL, emotional). Detailed instructions on scoring are provided. | No | No |
| **58** | Tinnitus Handicap Inventory (THI) | Newman, C. W., Jacobson, G. P., & Spitzer, J. B. (1996). Development of the Tinnitus Handicap Inventory. *Arch Otolaryngol Head Neck Surg,* 122(2), 143–148. doi: 10.1001/archotol.1996.01890140029007. | 1996 | Questionnaire | 25 | Pt are presented Qn about difficulties they may be experiencing because of their tinnitus. They are asked to answer each Qn with a 'yes', 'sometimes' or 'no' response. A severity scale is provided for interpretation (0-16=slight, 18-36=mild, 38-56=moderate, 58-76=severe and 78-100=catastrophic). | No | Yes |
| **59** | Tinnitus Handicap Questionnaire (THQ) | Kuk, F. K., Tyler, R. S., Russell, D., & Jordan, H. (1990). The psychometric properties of a tinnitus handicap questionnaire. *Ear Hear,* 11(6), 434–445. doi: 10.1097/00003446-199012000-00005. | 1990 | Questionnaire | 27 | Consists of statements about tinnitus. Pt are asked to indicate with a 0 ('strongly disagree') up to 100 ('strongly agree') their agreement with the statement. | No | No |
| **60** | Tinnitus Questionnaire (Hallam et al, 1988) | Hallam, R. S., Jakes, S. C., & Hinchcliffe, R. (1988). Cognitive variables in tinnitus annoyance. *Br J Clin Psychol,* 27(3), 213–222. doi: 10.1111/j.2044-8260.1988.tb00778.x. | 1988 | Questionnaire | 52 | Consists of statements about tinnitus. Pt are asked to score each with an A ('always'), B ('sometimes') or C 'never'. | No | No |
| **61** | Tinnitus Rating Scale (TRS) | Ahmed, M. F. ., & Khater, A. (2017). Tinnitus suppression after cochlear implantation in Pt with single-sided deafness. *Egypt J Otolaryngol,* 33(1), 61. doi: 10.4103/1012-5574.199404. | 2017 | Numeric rating scale | 5 | Pt are asked to rate their tinnitus on a 5-point Likert scale (1='not present' through to 5='present and debilitating'). | Not sure | No |
| **62** | Tinnitus Reaction Questionnaire (TRQ) | Wilson, P. H., Henry, J., Bowen, M., & Haralambous, G. (1991). Tinnitus reaction questionnaire: psychometric properties of a measure of distress associated with tinnitus. *J Speech Hear Res,* 34(1), 197–201. | 1991 | Questionnaire | 26 | Pt are presented with statements about effects of tinnitus on their lifestyle, general well-being etc. Pt are asked to circle a number on a 5-point Likert rating scale of 0 ('not at all') to 4 ('almost all of the time') that best describes how their tinnitus affects them. | No | No |
| **63** | Visual Analogue 6-point scale: Sound quality and annoying background noise were assessed using a six-item visual analogue scale (VAS), where 0 represented being unable to hear and 5 indicated hearing perfectly | Choi, J. E., Ma, S. M., Park, H., Cho, Y. S., Hong, S. H., & Moon, J. (2019). A comparison between wireless CROS / BiCROS and soft-band BAHA for Pt with unilateral hearing loss. *PLoS One,* 14(2), e0212503. doi: 10.1371/journal.pone.0212503. | 2019 | VAS | 2 | Consists of a 6-item VAS from 0 to 5, with 0='unable to hear' and 5='hearing perfectly'. Categories included Sound quality and Annoying background noise. | Not sure | No |
| **64** | Visual Analogue Scale (0-10 points): Subjects scored the loudness of the scale was assigned a score of 0 (no tinnitus), and the right-hand side of the scale was assigned a score of 10 (very loud, disturbing tinnitus). The subjects had to mark with an X where they perceived the loudness of their tinnitus to be | Van de Heyning, P., Vermeire, K., Diebl, M., Nopp, P., Anderson, I., & De Ridder, D. (2008). Incapacitating unilateral tinnitus in single-sided deafness treated by cochlear implantation. *Ann Otol Rhinol Laryngol,* 117(9), 645–652. doi: 10.1177/000348940811700903. | 2008 | VAS | 1 | Consists of a 10-point Likert scale from 0 to 10, with 0='no tinnitus' and 10='very loud, disturbing tinnitus'. Pt had to mark with an X where they perceived the loudness of their tinnitus to be. The VAS was completed for 2 conditions: with the CI activated, and with the CI deactivated. | Not sure |  |
| **65** | Visual Analogue Scale (1-10): Skin safety was evaluated by a visual analogue scale (between 1 and 10 from very bad to excellent) to rate cutaneous tolerance | Schmerber, S., Deguine, O., Marx, M., Van de Heyning, P., Sterkers, O., Mosnier, I., Garin, P., Godey, B., Vincent, C., Venail, F., Mondain, M., Deveze, A., Lavieille, J. P., & Karkas, A. (2017). Safety and effectiveness of the Bonebridge transcutaneous active direct-drive bone-conduction hearing implant at 1-year device use. *Eur Arch Otorhinolaryngol,* 274(4), 1835–1851. doi: 10.1007/s00405-016-4228-6. | 2017 | VAS | 1 | Skin safety was evaluated by the surgeon using a 1-10 VAS, with 1='very bad' to 10='excellent' rating of 'cutaneous tolerance'. Skin safety was evaluated up to 12 months post-op. | No | No |
| **66** | Visual Analogue Scale (VAS) | Pfiffner, F., Kompis, M., Flynn, M., Asnes, K., Arnold, A., & Stieger, C. (2011). Benefits of low-frequency attenuation of Baha® in single-sided sensorineural deafness. *Ear Hear,* 32(1), 40–45. doi: 10.1097/AUD.0b013e3181ecd002. | 2011 | VAS | 6 | Subjective sound quality was evaluated using questionnaires with an 11-point Likert scale from -5 to +5 VAS, rating brightness, softness, clarity, fullness, loudness (Ovegard et al. 1997), and reverberation. Two questionnaires were administered for each subject, one after completing the tests with the lowest cutoff frequency of 270 Hz, and the other after completing the tests with the highest cutoff at 1500 Hz. | Not sure | No |
| **67** | Visual Analogue Scale (VAS): An evaluation of the discomfort related to the possible tinnitus associated with the deafness will rely on a visual analogue scale (VAS) ranging from 0 to 10. This scale is presented as a 17 cm plastic ruler with a vertical arrow on one side and a graduated scale on the other side (0 to 10 cm). The subject first indicates the level of annoyance generated by the tinnitus on the vertical arrow using a cursor and the corresponding numeric value is reported by the evaluator. Then, the intensity of tinnitus is assessed using another ruler with the same dimensions | Marx, M., Costa, N. N., Lepage, B., Taoui, S., Molinier, L., Deguine, O., & Fraysse, B. (2019). Cochlear implantation as a treatment for single-sided deafness and asymmetric hearing loss: a randomized controlled evaluation of cost-utility. *BMC Ear Nose Throat Disord,* 19(1), 1. doi: 10.1186/s12901-019-0066-7. | 2019 | VAS | 2 | Discomfort related to the possible tinnitus associated with the deafness was measured on a VAS ranging from 0 to 10 presented as a 17 cm plastic ruler with a vertical arrow on one side and a graduated scale on the other side (0 to 10 cm). The subject first indicates the level of annoyance generated by the tinnitus on the vertical arrow using a cursor and the corresponding numeric value is reported by the evaluator. Then, the intensity of tinnitus is assessed using another ruler with the same dimensions. | Not sure | No |
| **68** | Visual Analogue Scale (VAS): Each day, they were asked to rate the loudness and stress caused by the tinnitus in a visual analogue scale, as well as their mood and their ability to influence the tinnitus. In the evaluation, visual analogue scale was translated to a scale from 0 to 10. The ratings were averaged for each subject over all 4 questions and each month | Buechner, A., Brendel, M., Lesinski-Schiedat, A., Wenzel, G., Frohne-Buechner, C., Jaeger, B., & Lenarz, T. (2010). Cochlear implantation in unilateral deaf subjects associated with ipsilateral tinnitus. *Otol Neurotol,* 31(9), 1381–1385. doi: 10.1097/MAO.0b013e3181e3d353. | 2010 | Diary | 4 | Pt rated loudness, stress caused by tinnitus, mood, ability to influence tinnitus on a VAS of 0-10 (higher numbers =better). Ratings were averaged over the 4 questions each month. | Not sure | No |
| **69** | Visual Analogue Scale (VAS): Subjective hearing handicap on a Visual Analogue Scale (VAS) 0-10 -Hearing handicap: as described by the experiencing of hearing problems in various everyday situations | Andersen, H. T., Schrøder, S. A., & Bonding, P. (2006). Unilateral deafness after acoustic neuroma surgery: subjective hearing handicap and the effect of the bone-anchored hearing aid. *Otol Neurotol,* 27(6), 809–814. doi: 10.1097/01.mao.0000227900.57785.ec. | 2006 | VAS | 1 | Pt are asked to rate their subjective hearing handicap in various everyday hearing situations on a VAS of 0 to 10. | Not sure | No |
| **70** | Visual Analogue Scale (VAS): Subjects were asked to mark the tinnitus severity on a 10-cm line anchored with the extreme labels “No tinnitus at all” and “Worst tinnitus imaginable.” | Galvin, J. J., Fu, Q. J., Wilkinson, E. P., Mills, D., Hagan, S. C., Lupo, J. E., Padilla, M., & Shannon, R. V. (2019). Benefits of cochlear implantation for single-sided deafness: Data from the House Clinic-University of Southern California-University of California, Los Angeles Clinical Trial. *EarHear,* 40(4), 766–781. doi: 10.1097/AUD.0000000000000671. | 2019 | VAS | 1 | Pt are asked to mark their tinnitus severity on a 10-cm line anchored with the extreme labels 'no tinnitus at all' and 'worst tinnitus imaginable'. | Not sure | No |
| **71** | Visual Analogue Scale (VAS): The VAS, assessing tinnitus loudness, is a simple ‘‘analogue’’ line, 10 cm in length, anchored by ‘‘quiet’’ and ‘‘very loud, cannot get any worse’’ | Mertens, G., Punte, A. K., De Ridder, D., & Van De Heyning, P. (2013). Tinnitus in a single-sided deaf ear reduces speech reception in the non tinnitus ear. *Otol Neurotol,* 34(4), 662–666. doi: | 2013 | VAS | 1 | Pt are asked to rate their tinnitus loudness using a simple 'analogue' line, 10 cm in length, anchored by 'quiet' and 'very loud, cannot get any worse'. The VAS score is determined by measuring in millimetres from the Lt-hand end of the line to the marked point. | Not sure | No |
| **72** | Visual Analogue Scale (VAS): The Visual Analogue Scale (VAS), assessing tinnitus loudness and disturbance, is a simple analogue line, 10 cm in length | Mertens, G., Hofkens, A., Punte, A. K., De Bodt, M., & Van de Heyning, P. (2015). Hearing performance in single-sided deaf cochlear implant users after upgrade to a single-unit speech processor. *Otol Neurotol,* 36(1), 51–60. doi: 10.1097/MAO.0000000000000653. | 2015 | VAS | 2 | Pt are asked to rate their tinnitus loudness and disturbance, on a simple analogue line, 10 cm in length, anchored by 'absolutely not' and 'absolutely'. The Lt-hand end represents 'complete disability' and the Rt-hand end 'complete ability'. | Not sure | No |
| **73** | Visual Analogue Scale (VAS): Tinnitus distress was measured with the visual analogue scale (VAS) before and 6 months after CI activation. The Pt had to mark the tinnitus strength on a scale from 0 (no tinnitus) to 10 (maximum strength) | Arndt, S., Aschendorff, A., Laszig, R., Beck, R., Schild, C., Kroeger, S., Ihorst, G., & Wesarg, T. (2011). Comparison of pseudobinaural hearing to real binaural hearing rehabilitation after cochlear implantation in Pt with unilateral deafness and tinnitus. *Otol Neurotol,* 32(1), 39–47. doi: 10.1097/MAO.0b013e3181fcf271. | 2011 | VAS | 1 | Pt are asked to rate their tinnitus distress on a VAS before and 6 months after CI activation. Pt had to mark the tinnitus strength on a scale from 0 ('no tinnitus') to 10 ('maximum strength'). | Not sure | No |
| **74** | Visual Analogue Scale (VAS): VAS assessment of tinnitus annoyance scores could range from 0 (not intense/not annoying) to 10 (intense/annoying) | Poncet-Wallet, C., Mamelle, E., Godey, B., Truy, E., Guevara, N., Ardoint, M., Gnansia, D., Hoen, M., Saaï, S., Mosnier, I., Lescanne, E., Bakhos, D., & Vincent, C. (2020). Prospective multicentric follow-up study of cochlear implantation in adults with single-sided deafness: Tinnitus and audiological outcomes. *Otol Neurotol,* 41(4), 458–466. doi: 10.1097/MAO.0000000000002564. | 2020 | VAS | 2 | Pt are asked to rate their tinnitus annoyance before and 13 months after surgery on a VAS ranging from 0 ('not intense/not annoying') to 10 ('intense/annoying'). | Not sure | No |
| **75** | World Health Organisation Quality of Life Short Form Survey (WHOQOL-BREF) | The WHOQOL Group (1998). Development of the World Health Organisation WHOQOL-BREF quality of life assessment. *Psychol Med,* 28(3), 551–558. doi: 10.1017/s0033291798006667. | 1998 | Questionnaire | 26 | Pt are presented with Qn about their QoL, health, and other areas of their life. Pt are asked to choose out of 5 answers which appears more appropriate. | No | No |
| **76** | Yes/No Answer to 'Beneficial to Hearing?' Question: Pt were asked whether they felt that the CROS amplification had been worthwhile and which of the three CROS devices (if any) took their preference | Hol, M. K. S., Kunst, S. J. W., Snik, A. F. M., & Cremers, C. W. R. J. (2010). Pilot study on the effectiveness of the conventional CROS, the transcranial CROS and the BAHA transcranial CROS in adults with unilateral inner ear deafness. *Eur Arch Otorhinolaryngol,* 267(6), 889–896. doi: 10.1007/s00405-009-1147-9. | 2010 | Single question | 1 | Pt are asked whether they felt the intervention has been worthwhile with 'Beneficial to Hearing?. A Yes/No answer was expected. | Not sure | No |
