# Supplementary material for: Assessment of current patient reported outcome measures for three core outcome domains for single-sided deafness device intervention trials
Source: J Patient Rep Outcomes. 2025 Jun 15;9:68. doi: 10.1186/s41687-025-00902-4 (PMC12167737; doi:10.1186/s41687-025-00902-4)
Supplement: Supplementary file 3 — Supplementary Material 3 [file 41687_2025_902_MOESM3_ESM.docx]

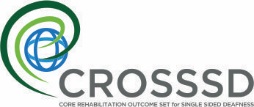


**Additional file 3.** Main discussion points and supporting participant quotes for the three outcome domains.

***Spatial orientation***

The functional domain topics derived for this outcome domain are: (i) being aware that sounds are not in your visual field, (ii) knowing where sounds are in relation to you, and (iii) attending to sounds in one location and not at other locations.

The main discussion points revolved around the presence of visual cues, which people need to rely on, to help them orientate in their environment.

[HU1] *“Where I always have a problem is basically where you can’t use your eyes to identify where a particular sound is coming from”*

[HP3] *“… have to turn their head all the time trying to oversee what’s around them”*

If visual cues are not available people often have to rely on other hints, such as others’ body language (e.g., where everyone else is looking) or assistance from others (e.g., a nudge from their partner).

[HU2] *“If I am sitting around a circular table, and someone addresses me, am often unaware of it, unless I can actually see who is addressing me; unless I see their body language or their face”*

[HP1] *“They say that they often get nudged or poked by the next person because they come in at the wrong time, or they miss something”*

Having an awareness that a sound (or a potential threat) is not in your visual field was also discussed, especially if it originates from behind.

[HP1] *“If you call for somebody or somebody talks to you or makes a sound and you don’t have them in your vision, you have no idea where that is coming from – that gives safety implications”*

[HP2] *“It’s not just about ‘can I turn left or right’ …, it’s the safety aspect, and your place in the world, which also influences your balance … your own orientation within space”*

Whether the sound is static (e.g., a barman calling) or moving (e.g., a moving car or an airplane) is also important.

[HU1] *“The most crucial thing. If I go into a house and call the name of the person I’d like to talk to I don’t know if they are upstairs or downstairs, I don’t know what room they are in”*

[HP1] *“Many patients like to hear the birds singing and they’ll say, ‘I can hear the bird singing, but I don’t even know which tree it’s in, let alone which branch in which tree’”*

Having an awareness that the sound is in a different space, and therefore does not require their attention or can be ignored helps people focus their attention accordingly and not get distracted.

[HU3] *“… it was a lot of people coming up behind me, it scared me because I didn’t know they were there”*

[HP1] *“… just the awareness that you get, the dynamic awareness that you get, when you are just moving around in spaces, not necessarily listening to a thing, or listening out to a thing …”*

The ability to quickly and accurately attend to sounds in environments with other distracting sounds, like background noise, without a time delay, was another important concept discussed.

[HU2] *“The background noise is a problem, because the background noise seems to for some reason take precedence. I can hear what is going on around me, the things I don’t need to hear I hear very clearly”*

[HU2] *“It’s quite difficult focusing onto the conversation that is going on in close proximity, because these other background sounds are very distracting”*

[HP1] *“I’ve had people walk, you know, go out with their CROS aid, and marvel at the fact that they are now able to walk past the waiting room and find that they can hear that there’s a noise there, even though they are not listening for it”*

Related to the main concepts, if people have a three-dimensional sense of their environment; they feel more comfortable, secure, and safe.

[HU4] *“… from behind in particular is quite scary because you don’t even have that visual cue or clue”*

Otherwise, if their environment is always unpredictable, they feel that they ‘miss out’, ‘stand out’, appear ‘vague’, can experience feelings of anxiety, inadequacy, frustration, stress, fear and feel that they are constantly challenged, which can make them ‘shy away’ from situations.

***Group conversations in noisy social situations***

The functional domain topics derived for this outcome domain are: (i) dynamic involvement, (ii) listening in the background of other conversations, and (iii) conversations in other background noise.

Discussions incorporated being able to dynamically participate in a conversation (i.e., move their attention quickly from one contributor to another), when someone new starts a new conversation thread.

[HU2] *“In a group situation, its knowing when I can interject; and learn when I can’t interject. Being aware of the fact that I have interjected when I shouldn’t have done, because there was still a conversation going on around me, and I hadn’t been aware of it, and that’s quite embarrassing. It’s very easy to retreat into a solitary existence; when it happens a couple of times, you feel you ought to, you know, step back and not get involved”*

It does not just include ‘listening’ and ‘following’ which are passive activities, but also ‘actively participating’ and contributing in conversations of groups constituting of three or more people.

[HU1] *“If there’s a group conversation with let’s say 4 or more, you don’t know when you can interject or something; and sometimes you might interject when someone is actually pausing, just to talk again. So I feel very, sort of, not very polite, actually talking over somebody”*

[HP2] *“It’s not just about ‘following a conversation’, because if you are following it you are always lagging behind, that would be being able to ‘part-take’ in a conversation”*

Maintaining attention for long enough can be influenced by the conversation partners. Family members can be more understanding and often choose more predictable topics.

[HP1] *“Defining a group is something about how many sets of conversations are going on … if there’s two conversations … and you get more of that as you get more people. So if you’ve got 4 people around the dinner table, often the conversation will be on one topic … if the conversations are too separate, that gets more challenging”*

However, it can be challenging to maintain conversations of a more unpredictable nature with new acquaintances or strangers who are unaware of the hearing impairment.

[HU1] *“When there’s 6 people having a conversation, there is a lot of cross-over conversations going. You know, one part if its talking about one subject, you can’t differentiate too easily between the two different conversations … I sometimes get very rude and I say: ‘can you just one person speak on one subject? And don’t keep moving?’ because I can’t then follow”*

[HP1] *“So it might be your wife and friend who have been in the conversation and suddenly they change topic and they are no longer in the conversation, they change from being conversation partners to ‘others’ in a second”*

The nature of background noise is also important: monotonous noise (e.g., a lawn mower) can be more easily ignored, speech sounds (e.g., children talking) are more difficult, and sudden unexpected sounds (e.g., a cup dropping, a child starting to cry) present the greatest challenge.

[HU4] *“Listening doesn’t always mean the same as understanding. And following. So I can be listening to people in a restaurant and not really understanding anything they are saying because I am listening but only getting little bits of it”*

[HU4] *“If there is any other sound in the room, it can ‘short circuit’ my concentration”*

[HP2] *“Speech is the hardest, if you’ve got competing speech all around you, that’s probably the hardest thing to filter out”*

When listening in the background of other conversations knowing what information is coming from your conversation as opposed to a conversation elsewhere, and focusing on the correct conversation are important elements to consider.

[HU2] *“People address me from various directions and I could be standing there, having a conversation, with maybe 1-2 people almost simultaneously and then a third or a fourth is interjecting, I find this a very challenging situation indeed”*

[HP1] *“There may be times when other people want to get your attention; and if you can’t tell where that is coming from, you can’t decide whether you are perfectly ok to ignore that interjection because it’s irrelevant to that time, or whether it’s important, or whether it’s a safety issue like someone is calling you because something is about to fall on your head; or, you have no idea, so your brain is always on high alert I think, to all possible sources of information”*

The listening environment has an impact. Social environments (e.g., a pub, a restaurant), or environments with poor acoustics (e.g., a large cathedral, an airport) can be detrimental. In these settings, people often do not have complete awareness of all conversations taking place and have to rely on visual cues, or non-verbal hints from conversation partners in order to contribute meaningfully at the right time.

[HU1] *“I won’t go out to restaurants, I don’t like noisy pubs”*

[HU3] *“It’s quite funny that you said ‘jokes’ cause I remember I was just laughing at random times, and it was only because I was like, putting people’s answers in their mouth for them. Because I was finding it funny and putting funny things in – I’d say ‘ha, didn’t you say that?’, and they went –‘no’. I was like ‘aw’. And that was quite weird. It happened quite a lot to be actually a thing that was quite funny in the end but quite embarrassing for me”*

People often feel that they are ‘being rude’, they get embarrassed, they have to be constantly on ‘high alert’ which can be tiring, can make them feel that they are not included in the group, they feel lonely and ‘retreat in solitary existence’ or ‘step back and not getting involved’.

[HU3] *“I could have been surrounded by all my best friends in a pub. And I would feel so lonely, because I couldn’t keep up. I didn’t know what was happening. I wouldn’t know where the conversation was going. I’d just be looking left and right. And it was quite sad*”

[HP2] *“You tend to withdraw, because it’s frustrating and demoralising isn’t it? And it is tiring”*

[HP3] *“Parties always are a problem, gatherings are a problem, and I think those who have problems with hearing in such situations they avoid those situations”*

***Impact on social situations***

The functional domain topics derived for this outcome domain are: (i) contributing to social interactions, (ii) ‘fitting in’ socially, and (iii) ease of participation.

The discussions concentrated on appropriate turn taking (i.e., knowing when it is appropriate to interject) and contributing to the social interaction with an appropriate interjection (e.g., comment on the correct topic). Accurate turn taking seems to be the key to ‘full participation’.

[HU1] *“I’d be very careful when there’s a group of people participating, to make sure I am fully aware of the subject they are discussing. Because I might miss some crucial points. Especially people who I don’t know really, because the people who I know would probably know my weaknesses and make allowanced for them”* ... *“It’d be more effort to keep up with a conversation like that, to make sure you don’t miss any particular subject. So you’d probably be using body language, eyes, a lot better that you’d normally do to actually try to ascertain what’s happening“ … “When someone is about to say something, you see them move. Or you see them move forward, with a group. So they are the sort of things you look for”*

[HP1] *“Whether people would engage or withdraw from social situations and there’s a lot of people who would say, ‘I don’t, I just don’t go out any more, or if I do, I don’t go out without my husband, or my wife, I have to make sure they always sit on my bad side because then I won’t get a stranger on my bad side and they know me so they can nudge me and, so yea, it’s those kind of things”*

The concepts discussed focused on the person with hearing loss being able to fully participate in a social situation, as opposed to just ‘listening’ and ‘following’ a conversation taking place with multiple people.

[HU4] *“I was not involved in the conversation. People at certain times were saying: ‘aw what do you think, or … and I’d just be like: ‘Can you tell me what you just said again please’? Because I had no idea what was happening in that conversation. So it’s quite lonely and it’s really sad when you have these situations”*

People often rely on conversation partners to inform them that the conversation has moved on to another topic (e.g., by repeating the last part of the conversation), or that someone else is still holding a turn. Inability to take correct turns (e.g., understanding and laughing at a joke at the right time) can make people feel that they are not an integral part of the social group.

[HP1] *“… about jokes and other new onsets of the conversation. You can follow a conversation maybe, but you don’t get the tiny little asides, or the little, the ‘hmhm’, that people say that re-affirm if it’s a positive or a negative or any of those things, or the new onsets, I forgot to mention earlier on, the ‘new onsets’ are the more challenging ones to get right. And maybe the more important ones to get right”*

Participating and being involved in social situations can be demanding and requires concentration and effort, therefore sustaining participation over a long period of time can be challenging.

[HP1] *“Many people, probably more people than in the usual hearing aid population are working-age people. And they’d be at work. And there will be a different dynamic in work. And meetings. And the, ah all of the fear about participation at work, whether this is about to influence your income and your lifestyle, so your single-sided or other deafness would have more of a direct impact that can’t really be got over by just your family members being kind to you”*

‘Over-participating’ (e.g., talking too much, or taking over the conversation) to compensate for the lack of awareness of correct turn-taking points, and also because listening and following a conversation is too demanding, was a related concept discussed.

[HP1] *“… your face, and your over-sharing, and over-participating. And if you don’t absolutely fit in the norms of what that conversation is about, then you’d stand out. And people will either look at you funny or they will tell you, or they will ignore it. But either way, you probably don’t feel that you have the same participation that other people might have”*

[HU2] *“I may be guilty personally sometimes of over-participating or trying to compensate for the deficiency in one area, which is the hearing loss, and perhaps maybe sometimes in the social situation is; speculating a bit too wildly possibly even speaking a bit too loudly, and not being aware at all of the fact that my behaviour, my speech, the volume, so on, the amplitude, are exaggerated. And that might sort of, you know, cause me to sort of, stand out and seem a bit sort of odd in a way”*

Being able to successfully keep up with conversations, gaining acknowledgement from others, and successful interactions, bring ‘involvement in life situations’ and lead to feelings of contribution and happiness.

[HU1] *“When you are in a social situation, and you are intensely listening to a conversation, that must come across weird to other people; you are almost screwing your face up listening to it. Equally it might not be socially acceptable”*

[HU2] *“I’m probably not alone in the world in wanting to conform, in wanting to appear to be normal. And you know, enjoy the benefit of, you know, accepted by my peers, and so on. So, yes, any way which this might be difficult to fulfil, you know, it’s kind of, it’s challenging, and stressful”*

[HU3] *“The whole reason why we socialise as human beings, to get a lot of things, back and forth a lot of acknowledgement, a lot of happiness”*

[HP2] *“The impact on social situations, it is how can you, ... your ability to take part in life basically”*

Otherwise, it can have an impact on relationships, participation in family, community, or societal situations and be correlated to participation restriction.

[HU2] *“I think I do become tired, you know, I lose the ability to sort of concentrate so greatly, and I do, after a euphoric few minutes I then turn sort of to take a back seat. And sit there, almost incapable”*

[HU3] *“People are just moody, they are just not, they just withdraw, and push back, because they are fed up of trying. And it’s, it can be quite traumatic … which I find quite, it’s quite daunting to me”*

[HP2] *“To fully participate in the social world it is about inclusion … there is this danger, if you have a hearing loss you withdraw from society … it’s got a massive impact”*

[HP3] *“The only thing you can do, is avoid these situations, or … be sure you are sitting on the right place on the table, close to the people you’d like to communicate with”*
